# Excited States of Bromopyrimidines Probed by VUV Photoabsorption Spectroscopy and Theoretical Calculations

**DOI:** 10.3390/ijms22126460

**Published:** 2021-06-16

**Authors:** Mónica Mendes, Fábris Kossoski, Ana I. Lozano, João Pereira-da-Silva, Rodrigo Rodrigues, João Ameixa, Nykola C. Jones, Søren V. Hoffmann, Filipe Ferreira da Silva

**Affiliations:** 1 CEFITEC, Departamento de Física, NOVA School of Science and Technology, FCT NOVA, Universidade NOVA de Lisboa, 2829-516 Caparica, Portugal; ai.lozano@fct.unl.pt (A.I.L.); jvp.silva@campus.fct.unl.pt (J.P.-d.-S.); rar09843@campus.fct.unl.pt (R.R.); j.ameixa@campus.fct.unl.pt (J.A.); f.ferreiradasilva@fct.unl.pt (F.F.d.S.); 2 Laboratoire de Chimie et Physique Quantiques (UMR 5626), Université de Toulouse, CNRS, UPS, CEDEX 09, 31062 Toulouse, France; 3 ISA, Department of Physics and Astronomy, Aarhus University, Ny Munkegade 120, 8000 Aarhus C, Denmark; nykj@phys.au.dk (N.C.J.); vronning@phys.au.dk (S.V.H.)

**Keywords:** VUV photoabsorption, halopyrimidines, valence and Rydberg states, time-dependent density functional theory, radiosensitizers

## Abstract

We report absolute photoabsorption cross sections for gas-phase 2- and 5-bromopyrimidine in the 3.7–10.8 eV energy range, in a joint theoretical and experimental study. The measurements were carried out using high-resolution vacuum ultraviolet synchrotron radiation, with quantum chemical calculations performed through the nuclear ensemble approach in combination with time-dependent density functional theory, along with additional Franck–Condon Herzberg–Teller calculations for the first absorption band (3.7–4.6 eV). The cross sections of both bromopyrimidines are very similar below 7.3 eV, deviating more substantially from each other at higher energies. In the 7.3–9.0 eV range where the maximum cross-section is found, a single and broad band is observed for 5-bromopyrimidine, while more discernible features appear in the case of 2-bromopyrimidine. Several π* ← π transitions account for the most intense bands, while weaker ones are assigned to transitions involving the nitrogen and bromine lone pairs, the antibonding σ*_Br_ orbital, and the lower-lying Rydberg states. A detailed comparison with the available photo-absorption data of bromobenzene is also reported. We have found significant differences regarding the main absorption band, which is more peaked in bromobenzene, becoming broader and shifting to higher energies in both bromopyrimidines. In addition, there is a significant suppression of vibrational structures and of Rydberg states in the pair of isomers, most noticeably for 2-bromopyrimidine.

## 1. Introduction

The absorption of ionizing radiation by living cells has long been recognized as a significant cause of long-term biological damage, leading to cellular death, mutations and/or cancer. Most biological effects of radiation arise from permanent modifications of the DNA structure, which can result in apoptosis [1]. It is well known that such consequences are mainly related to the effects of secondary low energy electrons and free radicals, which are produced by the interaction of the primary ionization radiation with the cellular molecular constituents [2,3]. Along the ionization track, secondary electrons are also able to excite biomolecular targets through inelastic collisions, thereby populating electronically excited states. These, in turn, might decay into further reactive species.

The ability of radiation in inducing modifications of the cellular components is particularly important and useful for treatment of diseases, especially in cancer therapy. Nowadays, the investigation and development of more efficient therapies based on the combination of radiation and chemotherapeutic agents represent an important and growing line of research. In chemoradiation, radiosensitizers are administered to the patient in order to enhance the radiosensitivity of tumor cells. In the subsequent radiotherapy stage, the damage is thus expected to be more localized in regions rich with radiosensitizers drugs. Through this combination, it is possible to increase tumor cell killing, with less damage to healthy tissues [4,5].

Pyrimidine is part of an important class of organic molecules, since it can be regarded as a building block of three nucleic acids, namely thymine, cytosine, and uracil. For this reason, its physicochemical properties have been studied through several experimental and theoretical techniques [6,7,8,9,10,11,12,13,14,15]. Chemical modification of pyrimidines by single halogenation, for example, has brought out their role as anti-cancer drugs. Indeed, it has been demonstrated that modified pyrimidines enhance the sensitivity of cells to ionizing radiation when incorporated into DNA as a substitute for thymine [16,17,18,19]. Halogenated analogues of thymidine, such as bromodeoxyuridine and iododeoxyuridine have shown great impact on the enhancement of the radiotherapeutic effects via the Auger electron cascade produced by the decay of the ionized heavy halogen atom [20,21,22]. Recently, some fluorine analogues, especially 5-fluorouracil, 5-fluorodeoxyuridine, have also demonstrated an important role in radiosensitization [5,23].

A full characterization of the electronic structure and electronic spectroscopy of pyrimidine and its derivatives is particularly relevant, to provide insights into photo-induced processes and their role in DNA damage. In particular, 2-bromopyrimidine (2BrPyr) and 5-bromopyrimidine (5BrPyr) are isomers generated by replacing a hydrogen atom by a bromine atom at positions C_2_ and C_5_ of pyrimidine, respectively. The effect of such substitutions in pyrimidine and pyrimidine-related compounds is important in order to better understand the site-specific chemical bonding in DNA/RNA molecules. The vibrational spectra of halogenated pyrimidines, including 2BrPyr and 5BrPyr, have been studied, with vibrational modes of different 2- and 5-monosubstituted pyrimidines [24,25]. Additionally, the vibrations of 2BrPyr and 5BrPyr were also investigated through infrared and Raman spectroscopy experiments [26,27,28].

Bolognesi and co-workers have performed an extensive experimental and theoretical analysis of halogen-substituted pyrimidines by X-ray photoemission spectroscopy [29], near edge X-ray absorption fine structure spectroscopy [30], photofragmentation by absorption of vacuum ultraviolet (VUV) and soft X-ray radiation [31,32], and Auger spectroscopy [33]. Castrovilli et al. [34] have measured the photoionization mass spectra of bromopyrimidines in the 9–15 eV energy range. The valence photoelectron spectra (PES) of halogenated pyrimidines, including 2BrPyr and 5BrPyr, were investigated by O’Keeffe et al. [35]. The authors observed that the halogen atom induces changes in the electronic structure of pyrimidine through two main mechanisms, the inductive and the resonant effect, which involve the π orbitals localized on the ring and the lone pair orbitals of the halogen atom. More recently, a higher resolution PES spectrum of 2BrPyr was reported by Śmiałek et al. [36]. Shape and core-excited resonances of halopyrimidines were investigated by electron transmission and dissociative electron attachment spectroscopies [37], as well as by electron scattering [38] and quantum chemical calculations [39]. Furthermore, the effect of bromination of thymine and uracil was the subject of investigations of electronic and vibrational excitation [40,41] and the formation of negative ions [41,42,43,44,45].

There is only one experimental investigation on the electronic excited states of 2BrPyr, by Joshi et al. [27]. They reported the VUV absorption spectrum (in arbitrary units of intensity), for photon energies ranging between 3.81 eV and 4.13 eV. VUV spectra were recorded in the gas-phase as well as in different solutions, with some vibrational assignments tentatively reported for the gas phase spectrum. This previous study was limited to lower energies and nothing is known about the higher-lying excited states of 2BrPyr. To the best of our knowledge, the excited states of 5BrPyr have never been studied.

In the present work, we investigate the electronically excited states of 2BrPyr and 5BrPyr, by means of experimental and theoretical methodologies. Specifically, we report high-resolution VUV absolute photoabsorption cross sections in the energy range of 3.7–10.8 eV, time-dependent density functional theory (TDDFT) calculations in combination with the nuclear ensemble approach (NEA), and Franck–Condon Herzberg–Teller (FCHT) calculations, for both bromopyrimidine isomers. Experimental and theoretical methodologies are explained in detail in Section 3. Our results bring forth a comprehensive picture of their excited states, and could shed further light on the subsequent production of radicals, which strongly impact the local site chemistry. Considering the radio-sensitizing potential of bromopyrimidines, the present data may also provide new insights on the effects of radiation damage at a nanoscale level and their role in chemoradiation applications.

## 2. Results and Discussion

**Figure 1:**
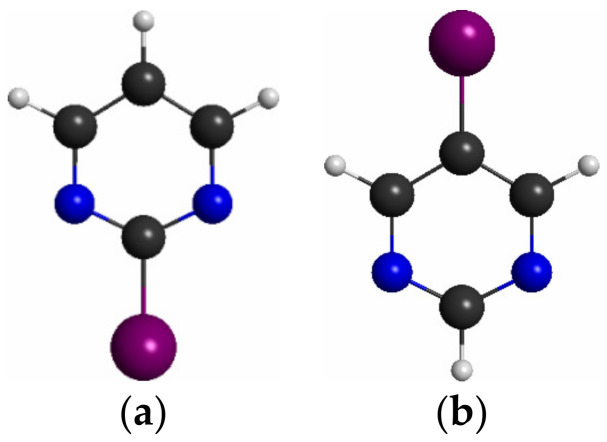
Molecular structure of (**a**) 2-bromopyrimidine, and (**b**) 5-bromopyrimidine. Carbon atoms in black, hydrogen in white, nitrogen in blue, and bromine in purple.

**Figure 2:**
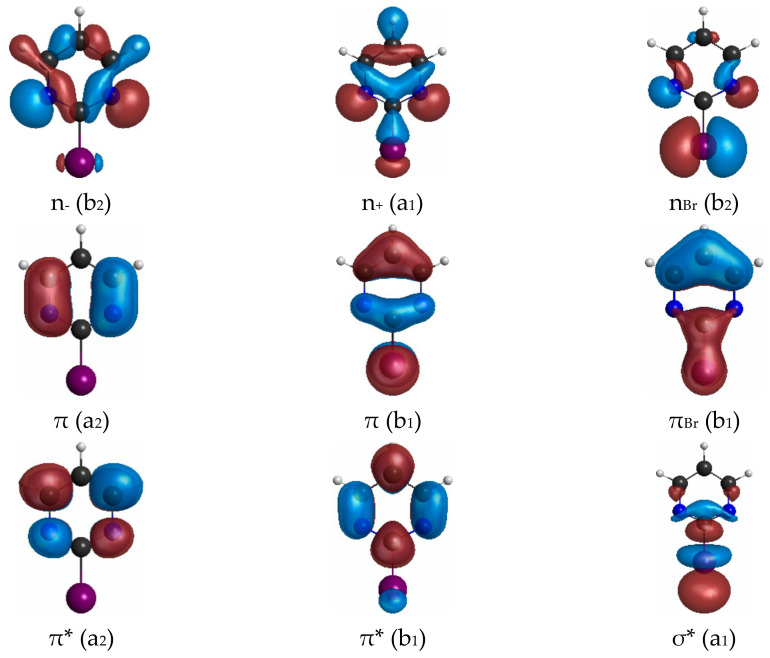
Typical hole and particle natural transition orbitals for the electronic transitions of 2-bromopyrimidine.

**Figure 3:**
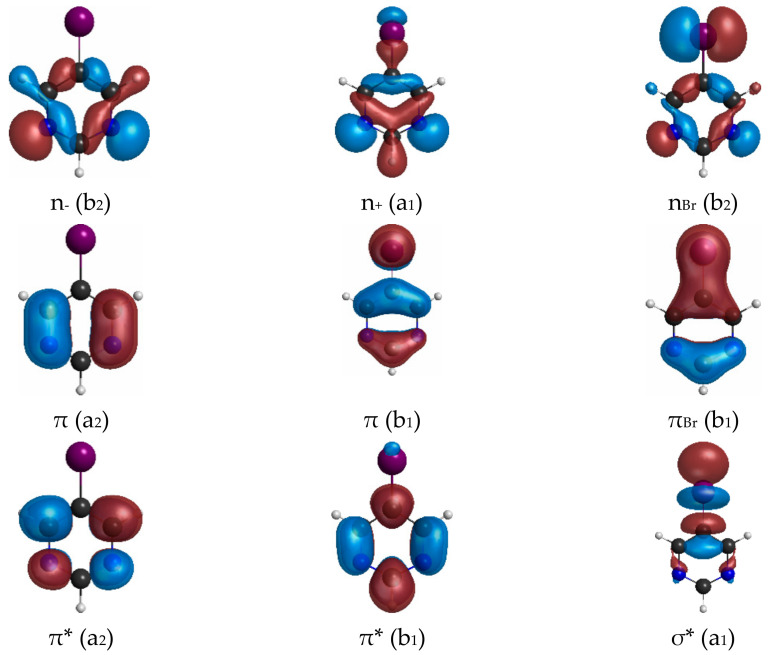
Typical hole and particle natural transition orbitals for the electronic transitions of 5-bromopyrimidine.

Both 2BrPyr (Figure 1a) and 5BrPyr (Figure 1b) belong to the C_2v_ point group. In the electronic ground state (A_1_ symmetry), the outermost molecular orbitals are … (18a_1_)^2^ (2a_2_)^2^ (5b_1_)^2^ (19a_1_)^2^ (10b_2_)^2^ (11b_2_)^2^ (6b_1_)^2^ for 2BrPyr, and … (18a_1_)^2^ (5b_1_)^2^ (2a_2_)^2^ (19a_1_)^2^ (10b_2_)^2^ (11b_2_)^2^ (6b_1_)^2^ for 5BrPyr, as obtained at the density functional theory/CAM-B3LYP/aug-cc-pVDZ + 2s2p2d level of theory. The frontier canonical orbitals are shown in the Supporting Information, in Figures S2 and S3. However, most electronic excitations involve more than one dominant pair of occupied and unoccupied canonical orbitals, making the interpretation of the transitions less straightforward. For this reason, we have also computed natural transition orbitals, which provide a compact representation of the dominant hole and particle orbitals that define the character of the transition [46]. While such orbitals are state-specific, they presented the same qualitative character along most of the excited states discussed here, and therefore our assignments are based on the set of typical natural transition orbitals displayed in Figure 2 and Figure 3. Most can be immediately associated with a corresponding canonical orbital: π*(a_2_) with the lowest unoccupied molecular orbital (LUMO), π*(b_1_) with the LUMO + 1, π(b_1_) with the highest occupied molecular orbital (HOMO), n_+_(a_1_) with the HOMO-3, π(a_2_) and πBr(b_1_) with either HOMO-4 or HOMO-5, depending on the isomer. In 5BrPyr, the n_-_(b_2_) natural transition orbital corresponds to the HOMO-1 and in 2BrPyr it arises as the combination of the HOMO-1 and the HOMO-2. The HOMO-2 of the former and the orthogonal combination of the latter provide the n_Br_(b_2_) orbital. Finally, the σ*_Br_(a_1_) orbital also appears in some excitations, and corresponds to higher-lying LUMOs.

**Figure 4:**
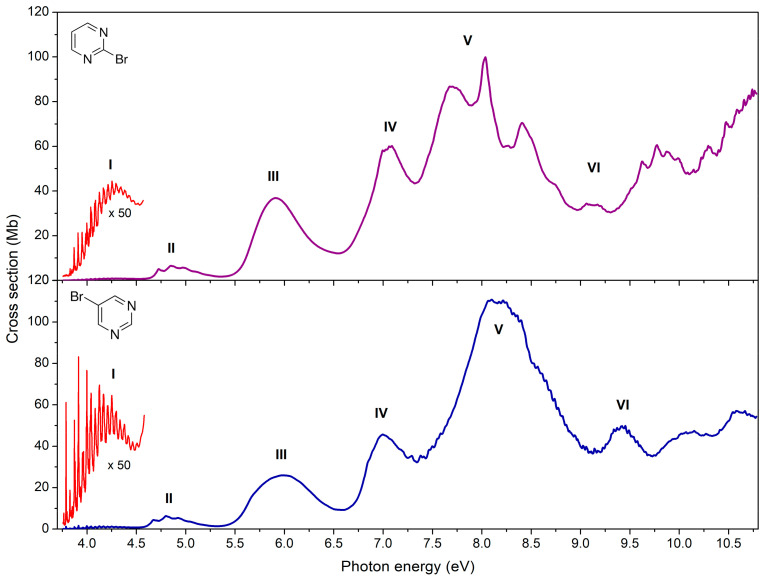
High-resolution VUV photoabsorption spectrum of 2-bromopyrimidine (**top** panel) and 5-bromopyrimidine (**bottom** panel) in the 3.7–10.8 eV photon energy range. Appropriate band labels (I–VI) are also given. See text for details.

**Figure 5:**
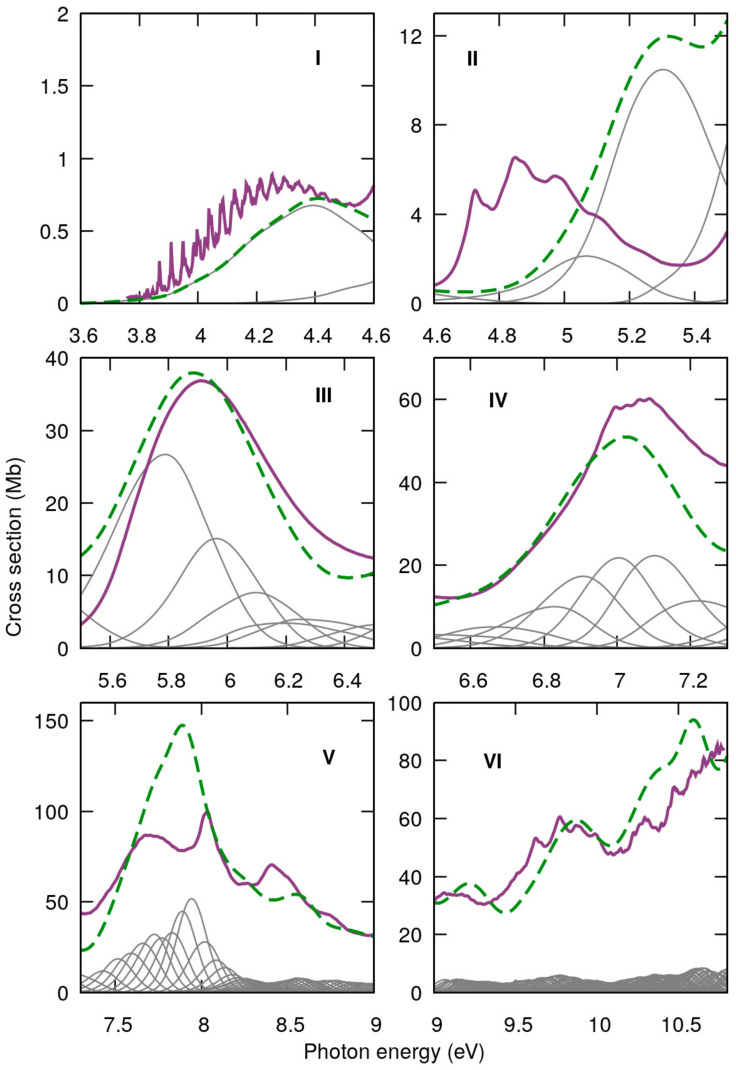
Comparison between measured (purple) and computed (green) absorption cross sections for 2-bromopyrimidine in the 3.7–10.8 eV photon energy range, separated into six absorption bands (**I**–**VI**). Individual contributions of the adiabatic excited states in grey.

**Figure 6:**
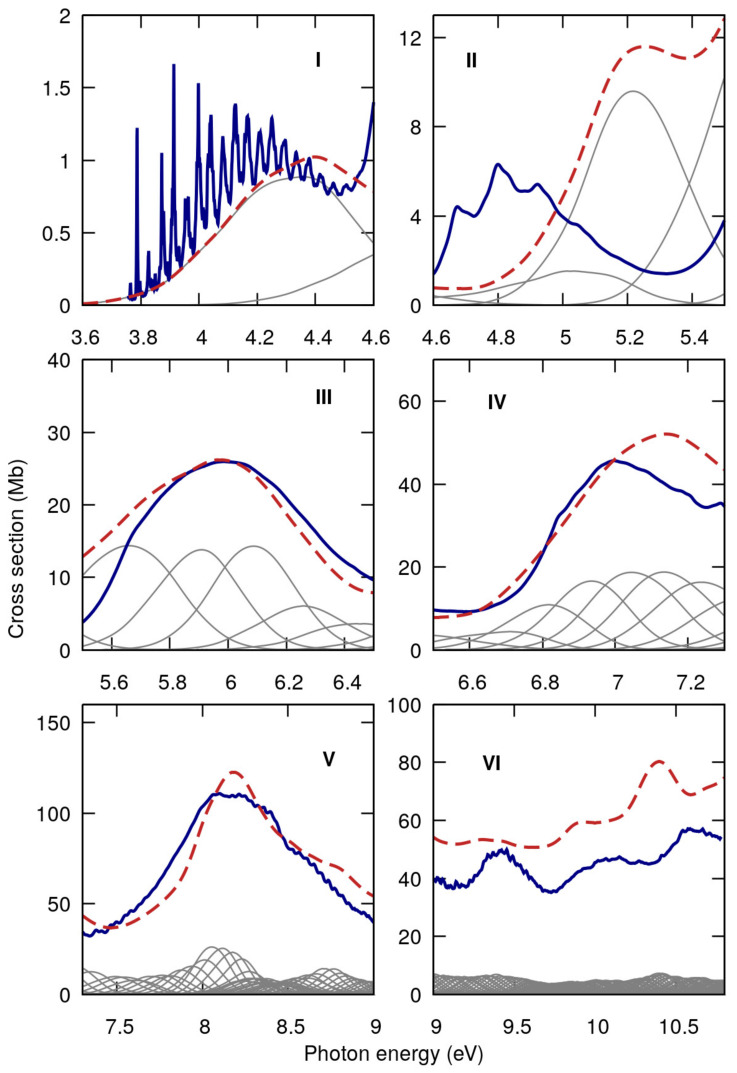
Comparison between measured (blue) and computed (red) absorption cross sections for 5-bromopyrimidine in the 3.7–10.8 eV photon energy range, separated into six absorption bands (**I**–**VI**). Individual contributions of the adiabatic excited states in grey.

Figure 4 shows the measured high-resolution VUV photoabsorption spectra of 2BrPyr (top panel) and of 5BrPyr (bottom panel) in the 3.7–10.8 eV photon energy range. Such high resolution measurements allowed us to resolve several fine features. The computed absorption cross sections are presented in Figure 5 and Figure 6, where they are compared with the experimental results. Agreement between experimental and theoretical cross sections are fairly good, both in magnitude and in shape. In light of this, we have interpreted the measured absorption bands largely based on the quantum chemical calculations. Table 1 and Table 2 summarize the computed vertical excitation energies and state characters, for each of the 40 lower-lying singlet excited states plus two higher-lying states presenting large oscillator strengths. The dominant character of the transitions was assigned by visually inspecting the corresponding natural transitions orbitals [46]. In Figure S1, we also compare the cross sections computed with the NEA method and with the simpler vertical approximation, which only takes the ground-state equilibrium geometry into account. Once the appropriate sampling of the ground state geometry is performed via the NEA method, the comparison with respect to the experiment improves significantly, for the whole energy range covered.

The VUV photoabsorption spectrum of each molecule shows an overall similar behavior for energies below 7.3 eV. The major differences between the two isomers are found at higher energies, most noticeably between 7.3 and 9.0 eV. The proposed vibrational assignments are based on the identification in the spectra of a given progression and its comparison to the computed vibrational frequencies, aided by inspecting the character of the vibrational modes and of the orbitals involved in the dominant excitations. The exception is Band I, where the assignments were performed more rigorously, based on a FCHT analysis. The vibrational modes and frequencies are presented in Tables S1 and S3, and were labelled according to the Mulliken convention.

Each of the following subsections is devoted to the discussion of a given absorption band (where energy ranges are loosely defined), while the last subsection concerns Rydberg states. In particular, the discussion will be focused on the effect of the position of the bromine atom on the VUV photoabsorption spectra, and we also compare with previous results for the closely related bromobenzene molecule [47], which are reproduced in Figure S4. A detailed comparison with pyrimidine [7] will be presented in a future paper.

### 2.1. The 3.7–4.6 eV Photon Energy Range (Band I)

**Figure 7:**
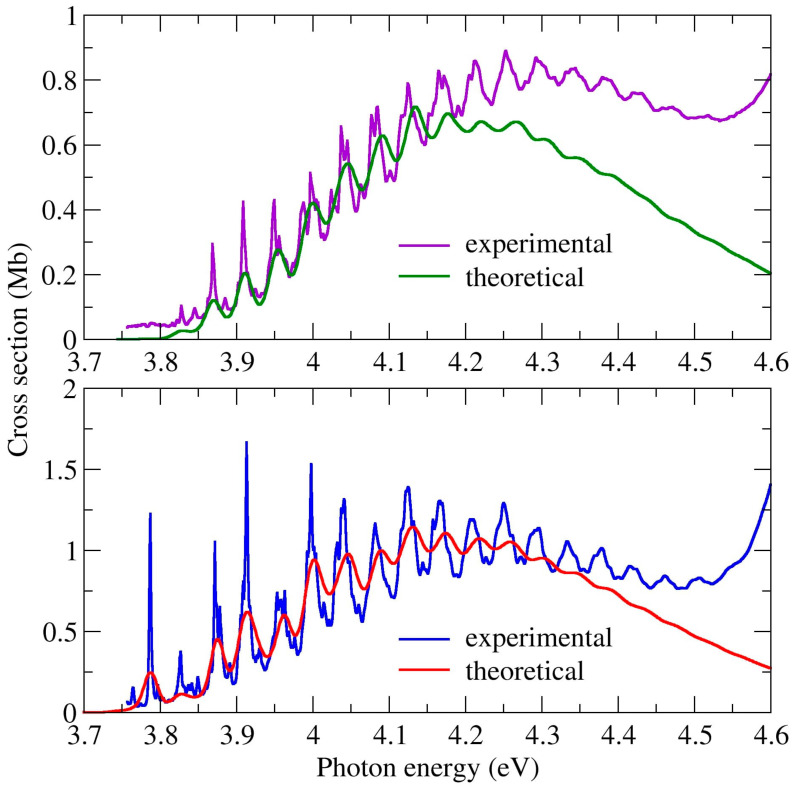
Comparison between measured and computed cross sections for the absorption Band I of 2-bromopyrimidine (**top** panel) and 5-bromopyrimidine (**bottom** panel).

In both isomers, the first absorption has an onset around 3.7 eV and extends up to 4.6 eV, as shown in Figure 7. Among all the bands discussed, this has the lowest intensities, yet the most fine structure. It is ascribed to the first singlet excited state, a π*(a_2_) ← n_-_(b_2_) transition. It involves excitation from the n_-_ non-bonding orbital of the nitrogen atoms, and thus has no parallel with bromobenzene [47].

This band is similar in both isomers, however in 5BrPyr it is somewhat more intense, slightly redshifted (~0.1 eV), and with more pronounced vibrational structures. The nuclear ensemble approach calculations reproduce very well the shape and magnitude of the bands, as well as the main observed differences between the two isomers. Importantly, the good agreement stems to a large extent from the ensemble averaging of the excitation energy, which decreases by 0.15 eV with respect to the vertical excitation energy, for both molecules. As pointed out before [48,49,50] this effect should be taken into account when comparing vertical excitation energies with the energy where absorption has a maximum.

**Figure 8:**
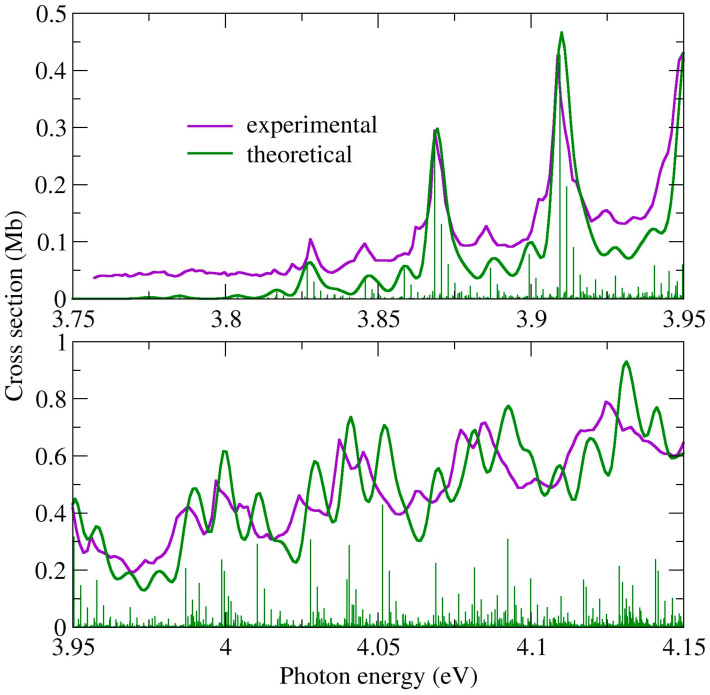
Comparison between measured and computed cross sections for the low energy tail of absorption Band I of 2-bromopyrimidine.

In 2BrPyr, the equilibrium geometry of the first singlet excited state (coordinates in the SI, and normal modes in Table S2) preserves the same symmetry operations as the ground state, and thus belongs to the C_2v_ point group. Therefore, there is a reasonable overlap between ground- and excited-state optimized geometries, and the underlying approximations of the FCHT analysis should hold. In Figure 7 we compare the measured and the computed absorption cross sections for the low energy tail of the band, between 3.75 and 4.15 eV. The theoretical curve was redshifted by 0.108 eV, in order to match with the observed 0–0 transition. In this comparison, we employed an arbitrary linewidth of 2.5 meV (~20.2 cm^−1^) for the FCHT calculations. Agreement is excellent, given the clear correspondence between most of the structures. This allows us to unequivocally pin down the band origin (the 0–0 transition) to the peak at 3.87 eV, which corresponds to the vibrational ground state of the excited state, thus agreeing with the assignment of Joshi et al. [27]. A number of low intensity hot bands are observed at lower energies, arising mostly from initially excited C-Br rocking (υ_24_) and stretching (υ_9_) modes. Towards higher energies, the peaks obtained with the FCHT analysis appear to be gradually blue shifted with respect to the measurements, which is an indication of further anharmonic effects not considered in the FCHT calculations. In fact, when they are performed without any anharmonic corrections for the excited state, such deviations are magnified. Eventually, the adiabatic Hessian approximation fails to reproduce the energies and shapes of the finer structures, especially above ~4.15 eV. Nevertheless, it still manages to capture the main observed vibrational progressions of the whole band, as shown in Figure 8, where a linewidth of 10 meV (~80.7 cm^−1^) was employed. A thorough assignment of the fine structures found in the 3.75–4.15 eV range can be found in Table S5. Despite the above described FCHT limitations, we also present in Table S6 the main transitions that should account for the main vibrational progressions up to ~4.1 eV. They comprise successive excitations of modes of a_1_ symmetry, mainly ring stretching modes having 700~1200 cm^−1^ frequencies and the C-Br stretching mode (see Table S6 for the full assignments).

For 5BrPyr, the calculations revealed a pair of degenerate equilibrium geometries for the first excited state (coordinates in the SI, and normal modes in Table S4), which arise from symmetry-breaking of the C_2_ axial symmetry. They still preserve a planar structure though, and thus belong to the C_s_ point group. Considering the existence of two equilibrium geometries and the smaller overlap between the lower-lying ground- and excited-state vibrational wave functions, the FCHT analysis is not expected to reproduce the fine structures observed for 5BrPyr. However, just as in the case of 2BrPyr, the calculations can account for the major vibrational progressions, as also shown in Figure 8 (the most intense computed transitions are presented in Table S7). Here a linewidth of 10 meV was employed, and the computed curve was redshifted by 0.097 eV. The very good comparison between the measured and the computed spectrum allowed us to identify the band origin at 3.79 eV. The main vibrational progressions arise from successive excitations of ring stretching modes presenting frequencies in the 700~1200 cm^−1^ range, and also of the C-Br stretching mode, which overall share the same characters as those identified for 2BrPyr.

### 2.2. The 4.6–5.4 eV Photon Energy Range (Band II)

**Figure 9:**
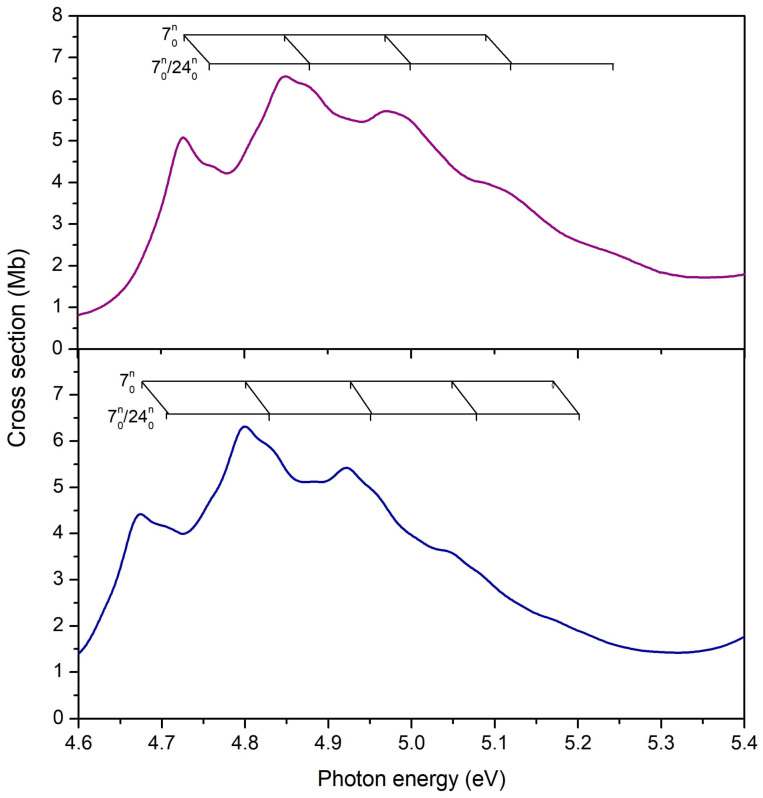
Vibrational progressions in the 4.6–5.4 eV energy range of the absorption Band II of 2-bromopyrimidine (**top** panel) and 5-bromopyrimidine (**bottom** panel).

Figure 9 shows the second absorption band, found between 4.6 and 5.4 eV. Their magnitude and shape are very similar in both molecules, yet they appear displaced with respect to each other by around 0.05 eV, being lower in 5BrPyr. We assign this band to excitation of the third excited state, a π*(a_2_) ← π(b_1_) dipole allowed transition, though the second excited state, a dipole forbidden π*(b_1_) ← n_-_(b_2_) transition, contributes to a lesser extent. The band origin 0–0 is tentatively placed at 4.72 eV for 2BrPyr and at 4.68 eV for 5BrPyr, whereas the observed vibrational progressions of both molecules are assigned to ring stretching (υ_7_) and Br rocking (υ_24_) modes (see Table 3 and Table 4). The present calculations overestimate the cross sections by a factor of two, and place the band too high in energy by around 0.5 eV, for both isomers. This disagreement stands out from the much better comparison observed for the other bands. Going from the vertical approximation to the NEA results does not bring any improvement (see Figure S1), and therefore, the problem should be related to the choice of functional (CAM-B3LYP), while basis set effects proved to have a minor role. We notice, though, that the π(b1) and π*(a2) orbitals also take part in other transitions where the comparison with experiment is more favorable. Thus, it is not clear why the theoretical description of this particular band falls short.

In bromobenzene, the analogous π*(a_2_) ← π(b_1_) state was also found in the same energy range, where it accounts for the first absorption band [47]. There are, however, two key differences. First, the cross sections are considerably larger in the bromopyrimidines (having a maximum of 6–7 Mb) than in bromobenzene (around 1 Mb). Second, the vibrational structures are much narrower in the latter, pointing to considerably longer-excited state lifetimes. In this energy range, the photodissociation mechanism of bromobenzene has been suggested to involve intersystem crossings from the bright singlet state to dissociative triplet states [51,52]. Assuming a similar mechanism takes place in the bromopyrimidines, significantly larger intersystem crossing rates from the π*(a_2_) ← π(b_1_) state would be expected.

### 2.3. The 5.4–6.5 eV Photon Energy Range (Band III)

The next absorption band lies between 5.4 and 6.5 eV, peaking at 5.91 eV for 2BrPyr and at 5.98 eV for 5BrPyr. In neither are there distinct vibrational structures (see Figure 4). The band profile is similar in both molecules, though the magnitudes differ from a maximum of 37 Mb in 2BrPyr to 26 Mb in 5BrPyr. The theoretical calculations support that the bulk of the intensity is due to the π*(b_1_) ← π(b_1_) transition, which have comparable excitation energies and a somewhat larger oscillator strength in 2BrPyr.

We notice, however, a subtle difference between the two spectra. It is more peaked in 2BrPyr and asymmetric in 5BrPyr, where a shoulder around 5.7 eV can be discerned. Considering that the NEA calculations seem to reproduce this feature, it might have an electronic origin, rather than a vibrational one. In fact, while other excitation energies in this range are rather close in both molecules, the σ*_Br_(a_1_) ← π(b_1_) transition appears 0.45 eV lower in 2BrPyr than in 5BrPyr. Such a big shift would arise from the σ*_Br_(a_1_) orbital, as discussed in the context of negative ion states [34,35,36]. The ensemble averaging further decreases the excitation energies by 0.25 eV (in both molecules), besides promoting intensity borrowing from the bright state. The greater sensitivity of this state on the position of the bromine atom could thus explain the observed difference in the shape of this band.

In the case of bromobenzene, the analogous band emerges in the same energy range, also stemming from a dominant π*(b_1_) ← π(b_1_) transition [47]. Moreover, the vibrational profile observed for bromobenzene is completely lost in the bromopyrimidines. In the latter, the σ*_Br_(a_1_) ← π(b_1_) excited state (dissociative along the C-Br bond) lies within the band, whereas in the former the analogous state arises in the high energy tail of the band. Therefore, more favorable couplings between the optically bright state and the dissociative state are expected in the bromopyrimidines, which could explain the lack of vibrational structures.

### 2.4. The 6.5–7.3 eV Photon Energy Range (Band IV)

**Figure 10:**
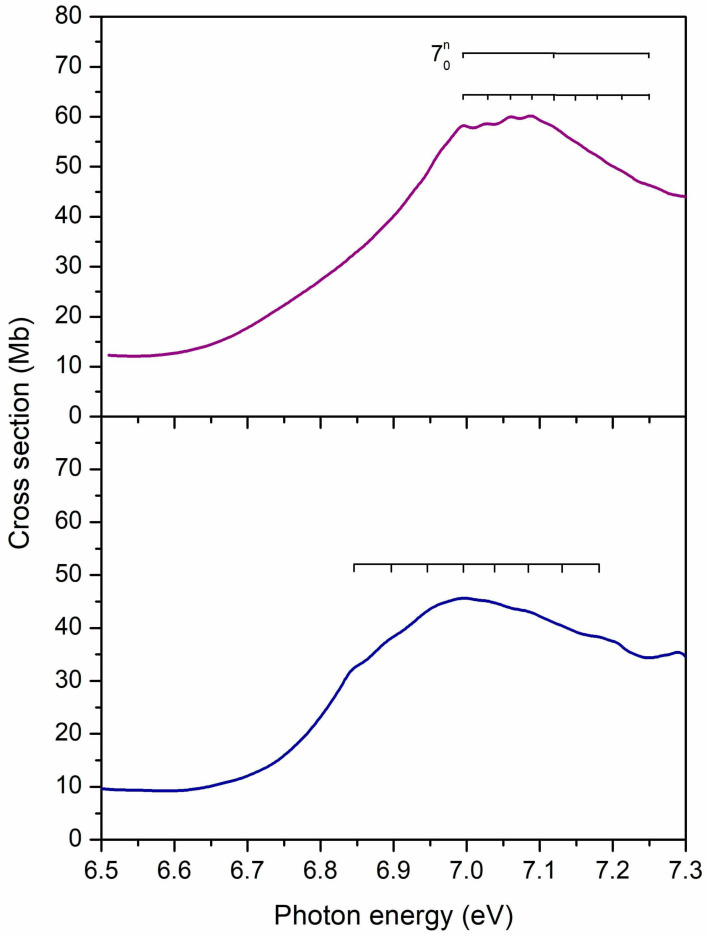
Vibrational progressions in the 6.5–7.3 eV energy range of the absorption Band IV of 2-bromopyrimidine (**top** panel) and 5-bromopyrimidine (**bottom** panel).

This energy range accommodates the fourth absorption band (see Figure 10), having a maximum at 7.09 eV in 2BrPyr, and at 7.00 eV in 5BrPyr. In both, the π*(a_2_) ← π(a_2_) transition brings most of the intensity to this band, even though other excitations also contribute to some extent, in particular the 3s(a_1_) ← π(b_1_) transition for 2BrPyr, and the π*(b_1_) ← π(a_2_) + π*(a_2_) ← π(b_1_) transition for 5BrPyr. Differences in the characters and oscillator strengths of such secondary excitations could explain the observed differences found in the vibrational progressions of each spectrum. In 5BrPyr the band origin 0–0 is tentatively placed at 6.84 eV, and at 6.99 eV for 2BrPyr. The latter develops into two sets of progressions, one has an average energy spacing of 0.13 eV and is tentatively assigned to a ring stretching (υ_7_) mode. The other has an average energy spacing of 0.03 eV, and also seems to be present in 5BrPyr, though with an observed spacing of ~0.05 eV. A close inspection of Tables S1 and S3 reveals that no computed mode matches the observed ones. This may indicate that a significant relaxation of the excited states takes place, resulting in rather different frequencies.

In bromobenzene [47], the most intense band of the VUV spectrum is in the 6.3–7.0 eV range, being redshifted by around 0.3 eV with respect to the Band IV of the bromopyrimidines, besides being considerably more intense. As will be clearer in the next section, the intense band of bromobenzene should not only be compared with Band IV of bromopyrimidines, but also with part of Band V. In bromobenzene, the band has been assigned to two major excitations, the dominant one with A_1_ symmetry and π*(a_2_) ← π(a_2_) + π*(b_1_) ← π(b_1_) character, followed by the π*(b_1_) ← π(a_2_) + π*(a_2_) ← π(b_1_) excitation, of B_2_ symmetry. Except for the leading term of the former excitation, the other terms are absent in the Band IV of bromopyrimidines, giving rise to the next band, as discussed below.

### 2.5. The 7.3–9.0 eV Photon Energy Range (Band V)

**Figure 11:**
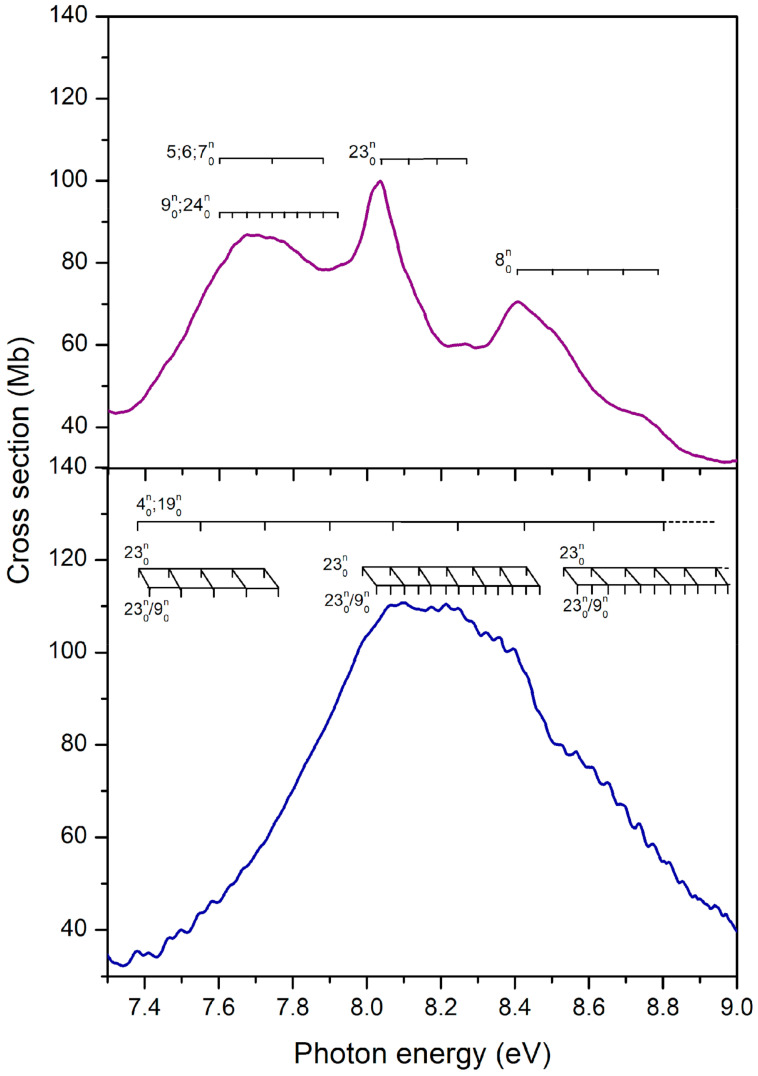
Vibrational progressions in the 7.2–9.0 eV energy range of the absorption Band V of 2-bromopyrimidine (**top** panel) and 5-bromopyrimidine (**bottom** panel).

The most intense absorption band lies between 7.3 and 9.0 eV, for both bromopyrimidines, where several optically bright excited states are involved (see Figure 5, Figure 6 and Figure 11). In contrast to the four previous bands, here the position of the bromine atom greatly affects the behavior of the cross sections, which present many more discernible features in 2BrPyr, whereas in 5BrPyr the band is considerably broader. The latter also presents overall larger magnitudes and much more pronounced vibrational progressions. Indeed, due to the complexity of the spectra in this energy region, and the fact that some vibrational modes have close frequencies, our vibrational assignments become more tentative for this band.

In 5BrPyr, the band is centered at 8.2 eV, and is assigned to π*(a_2_) ← π_Br_(b_1_) + π*(b_1_) ← π(a_2_) (f_0_ = 0.32) and π*(b_1_) ← π_Br_(b_1_) (f_0_ = 0.15) excitations, vertically located at 8.18 and 8.28 eV according to the calculations. Two vibrational progressions were identified and tentatively attributed to excitation of the C–Br stretching (υ_9_) and ring stretching (υ_23_) modes (see Table 5). Another progression with an average spacing of 0.180 eV (1452 cm^−1^) was also identified, and could be related to either υ_4_ or υ_19_ ring stretching modes.

The analogous band of 2BrPyr presents a broader peak centered at 7.67 eV, followed by a sharp peak at 8.05 eV. A quick inspection of Table 1 indicates three states (vertically at 7.75, 7.87, and 8.00 eV) with large oscillator strengths, having 3p_x_(b_1_) ← π(b_1_) (f_0_ = 0.19), π*(b_1_) ← π_Br_(b_1_) (f_0_ = 0.22), and π*(b_1_) ← π(a_2_) + π*(a_2_) ← π_Br_(b_1_) (f_0_ = 0.43) characters. While these states should account for the two observed features, we cannot be sure which ones contribute to which peak, in view of their close-lying energies. Two vibrational progressions were tentatively assigned to ring stretching (υ_5_, υ_6_, υ_7_) and Br rocking (υ_24_) or C-Br stretching (υ_9_) modes. At higher energies, a ring deformation (υ_23_) and a ring stretching (υ_8_) were also tentatively assigned (see Table 6).

Clearly, the brightest electronic transitions in both of the bromopyrimidine isomers share the same character, and have similar excitation energies and oscillator strengths. The main contributions concern excitations of π*(a_2_) ← π_Br_(b_1_), π*(b_1_) ← π(a_2_), and π*(b_1_) ← π_Br_(b_1_) characters, which account for the large intensity of Band V. The remaining excitation involving these orbitals, π*(a_2_) ← π(a_2_), is the one responsible for Band IV, as discussed above. Recalling that the same four excitations give rise to the intense absorption peak around 6.3–7.0 eV in bromobenzene [47], we may conclude that the analogous band in both bromopyrimidines is displaced to higher energies, becomes considerably broader, and actually splits into more discernible features. In this sense, the third and most intense band in bromobenzene would correspond to the combination of Band IV and the first half of Band V in the bromopyrimidines, extending over the 6.8–8.2 eV energy range in 2BrPyr and the 6.8–8.5 eV range in 5BrPyr.

Embedded into the broad Band V, the distinct peak at 8.41 eV in 2BrPyr as well as the broad shoulder around 8.7 eV in 5BrPyr arise from σ*_Br_(a_1_) ← n_+_(a_1_) + 3p_y_(b_2_) ← n_Br_(b_2_) transitions. The more stable σ*_Br_(a_1_) orbital of 2BrPyr explains the observed redshift for this transition, similarly to what we have discussed for the Band III.

### 2.6. The 9.0–10.8 eV Photon Energy Range (Band VI)

The energy region between 9.0 and 10.8 eV presents no sharp peaks as the cross sections vary less abruptly, displaying instead some broader and several narrow features. Many valence and Rydberg excited states can be populated in this energy regime, which may couple among themselves and develop complicated vibrational progressions, making interpretation of the spectra especially difficult. Despite that, we hope our TDDFT and NEA calculations can provide at least qualitative results at these energies. In fact, the computed cross sections are quite comparable to the measured ones for 2BrPyr, though somewhat overestimated for 5BrPyr. Importantly, the major features of both molecules are correctly reproduced by the calculations.

**Figure 12:**
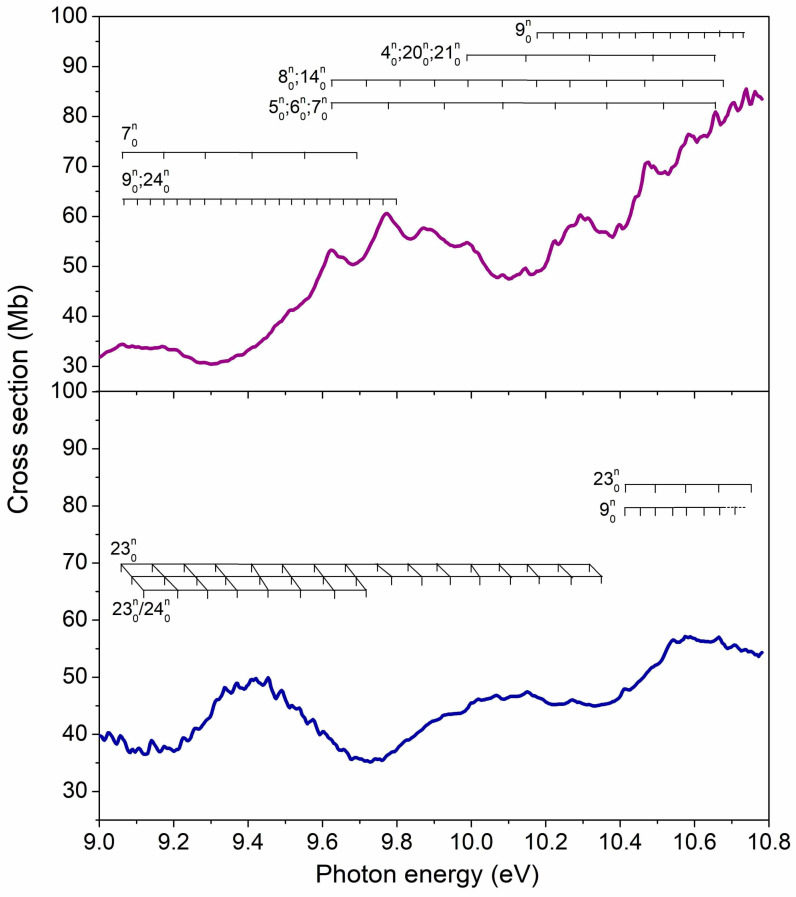
Vibrational progressions in the 9.0–10.8 eV energy range of the absorption Band VI of (**top** panel) and 5-bromopyrimidine (**bottom** panel).

A close inspection of Figure 12 shows some differences between the two spectra. Three vibrational progressions were identified for 5BrPyr and were tentatively attributed to excitation of the in-plane Br rocking (υ_24_), a ring stretching (υ_23_), and the C-Br stretching (υ_9_) modes (see Table 7). In 2BrPyr, the vibrational assignments were more challenging, and different vibrational modes were identified (see Figure 12), as summarized in Table 8.

In comparison to bromobenzene [47], the cross sections of both bromopyrimidines are smaller in this energy range, besides having less pronounced features. Both observations could be related to the suppression of Rydberg states, discussed in the next section.

### 2.7. Rydberg States

Rydberg states are characterized by excitation into diffuse (Rydberg) orbitals, whose energies follow the well-known Rydberg formula, *E_n_* = IE − [13.606/(*n* − *δ*)^2^] [eV], where the excitation energy *E_n_* is estimated from the ionization energy IE, the principal quantum number *n*, and the quantum defect *δ*. For each angular momentum (s, p, d, etc.), and for each orbital from which the electron is excited, the Rydberg states form a series converging to the ionization energy associated with this orbital. Such states tend to appear in VUV spectra as sharper peaks than valence excitations, though with varying intensities, and their precise assignment is usually performed with the aid of the Rydberg formula.

Our measured spectra do not display pronounced signatures of Rydberg states, for either 2BrPyr and 5BrPyr, in marked contrast to what was observed for bromobenzene [47]. That being said, there is some evidence that weak structures in the VUV spectra would arise from the 3s(a_1_) ← π(b_1_) Rydberg state. In 2BrPyr, the four small peaks observed in Band IV (starting at 6.99 eV) could actually correspond to a vibrational progression of this Rydberg state. The theoretical calculations further support this assignment, as this state was found at 7.00 eV, with an oscillator strength of 0.045, which, albeit small, is probably sufficiently large to leave a weak mark on the spectrum. The associated quantum defect would be 0.84, considering the experimental ionization energy of 9.93 eV for the π(b_1_) orbital [36]. In 5BrPyr, the analogous 3s(a_1_) ← π(b_1_) Rydberg state could account for the weak shoulder observed at 6.84 eV (a quantum defect of 0.90, given an ionization energy of 9.93 eV [35]). Again, this is supported by the calculations, which show an excitation energy of 6.89 eV, and an oscillator strength of 0.016. In particular, the lower oscillator strength in 5BrPyr is also consistent with the weaker feature on the spectrum.

According to our calculations, some of the 3p Rydberg states presented strong mixing with valence states, contributing to Band V. In contrast, purer 3p Rydberg states that show up in the same energy range (7.5–8.0 eV) have reduced oscillator strengths, thus leaving no distinct signatures in the spectrum. Finally, the 3d Rydberg states would start emerging in the 8.0–8.5 eV range, most of them with low intensities.

Towards higher energies, a multitude of Rydberg series are expected, though with progressively smaller intensities. In fact, the theoretical calculations (see Table 1 and Table 2) indicate that Rydberg transitions become more and more prevailing at higher energies, though with small oscillator strengths, particularly for 2BrPyr. For instance, the 4s(a_1_) ← π(b_1_) state of this isomer would appear at 8.48 eV according to our calculations, with a very low oscillator strength (0.001). The same applies for the other angular momentum and for excitations from other high-lying occupied orbitals. Indeed, at higher energies we have observed no sharp peaks that could be clearly associated with Rydberg states. Rather, the absorption spectra present a large number of closely-lying weak features. Thus, any attempt to locate the Rydberg series based on the Rydberg formula and the ionization energies would be very tentative and not conclusive. To a great extent, that would be a less pertinent task, considering the overall minor contribution of the higher-lying Rydberg states to the photoabsorption cross section of bromopyrimidines.

The scenario is quite distinct for bromobenzene, where much more pronounced structures have been observed [47]. With the support of high-resolution PES data, this allowed several Rydberg states to be clearly assigned. We notice that a similar behavior can be recognized when one compares the VUV spectra of benzene and pyrimidine [7,53]. Such observations suggest that going from benzene to pyrimidine, as well as from bromobenzene to bromopyrimidines, promotes a major suppression of the Rydberg series.

## 3. Methods

### 3.1. Experimental Methods

The high-resolution VUV photoabsorption measurements were performed at the AU-UV beamline of the ASTRID2 synchrotron facility at Aarhus University in Denmark. A detailed explanation of the experimental apparatus is given by Palmer et al. [54] and an in-depth description of procedures for measurement and calibration can be found in Duflot et al. [55]. In short, the set-up consists of a gas cell placed on the output of the monochromator where a MgF_2_ window was mounted to separate it from the ultra-high vacuum (UHV) of the beam line. The synchrotron radiation passes through a static gas sample and the transmitted light intensity is detected by a photomultiplier tube (PMT). The absolute pressure of the effusive molecular gas is measured with a capacitance manometer (Chell CDG100D). The wavelength is selected using a toroidal dispersion grating with 2000 lines/mm providing a resolution of 0.075 nm, corresponding to 3 meV at the midpoint of the energy range studied. In order to avoid any absorption of O_2_ from the air in measurements below 200 nm (energies above 6.20 eV), the small gap between the PMT and the MgF_2_ gas cell exit window is evacuated using a scroll pump. For higher wavelength measurements, air is admitted into the gap at atmospheric pressure to allow O_2_ to absorb any higher orders of light produced by the monochromator. ASTRID2 operates in “top-up” mode, keeping the stored electron beam current quasi-constant. Absolute photoabsorption cross sections (σ) were obtained at room temperature (~25 °C) by using the Beer−Lambert attenuation law (1),

*I_t_* = *I*_0_ exp(−*Nσl*), (1)

where *I_t_* is the light intensity transmitted through the gas sample, *I*_0_ is that transmitted through the evacuated cell, *N* is the molecular density, and *l* is the absorption path length (15.5 cm). The range of pressures used for the measurements of 5BrPyr was 0.01 to 0.54 mbar, while for 2BrPyr, which has a lower vapor pressure, the pressure range was 0.02 to 0.11 mbar. The accuracy of the cross section is estimated to be ~10%, which is ensured by recording the VUV spectra in small (5 or 10 nm) sections to allow optimization of pressure according to the local cross-sections, with at least 10 points overlap between the adjoining ranges [55,56].

The 2-bromopyrimidine sample was purchased from FluoroChem with a stated purity of ≥97%, while 5-bromopyrimidine was purchased from Sigma Aldrich with a stated purity of ≥97%. The samples were used with no further purification and degassed before measurement through repeated freeze−pump−thaw cycles.

### 3.2. Theoretical Methods

The photoabsorption cross sections were computed with the NEA [57,58] in combination with TDDFT calculations. The latter were performed with the Gaussian 16 software [59], while the managing of the NEA calculations were performed as implemented in the Newton-X package [60,61].

We employed the CAM-B3LYP functional, which provided excitation energies comparable to those observed in the measured VUV spectra. Other range separated functionals were explored (LC-ωPBE, ωB97X, and LCBLYP), however overall, they did not perform as well as CAM-B3LYP for the cases of 2BrPyr and 5BrPyr. We used the aug-cc-pVDZ basis set supplemented with a set of 2s2p2d diffuse functions, centered at the carbon atom closest to the bromine atom. For each angular momentum, the exponents were generated by successive divisions of the previous one by four (starting from the most diffuse functions of the aug-cc-pVDZ set). The CAM-B3LYP/aug-cc-pVDZ+2s2p2d level of theory was employed for the geometry optimization, normal mode analysis, and calculation of anharmonic frequencies (see below).

In the NEA, the configurations are sampled according to the Wigner distribution (Gaussians) of a set of independent harmonic potentials, which represent the normal modes at the ground state optimized geometry. The corresponding Wigner distribution at 298 K (experimental condition) was considered for the sampling. Anharmonic corrections for the frequencies were accounted for with the importance sampling algorithm [62], though such effects had a marginal impact on the computed cross sections.

Excitation energies Δ*E*0*n*(*q_i_*) and oscillator strengths f_0*n*_(*q_i_*) of the *n*-th excited state are computed for each sampled configuration *q_i_*. Then, the photoabsorption cross section is obtained as (in atomic units):

(2) $$\sigma \left(E\right)=\frac{1}{E}\;\sum_{n}^{N_{s}}\frac{1}{N_{p}\left(n\right)}\sum_{i}^{N_{p}}\Delta E_{0n}\left(q_{i}\right)f_{0n}\left(q_{i}\right)g\left[E-\Delta E_{0n}\left(q_{i}\right),\delta \right],$$

where the phenomenological line profile *g* is represented by a normalized Gaussian function centered at each excitation energy Δ*E*_0*n*_(*q_i_*). Here we have employed a linewidth of *δ* = 0.1 eV, which is large enough to damp the artificial undulations of the cross-section curve, yet small enough to keep its actual overall shape. We have used a decreasing number of configurations as we moved higher in energy. For both 2BrPyr and 5BrPyr, the number of sampled configurations was *N_p_* = 7500 for the first eight singlet excited states (*n* = 1–8), *N_p_* = 5500 for *n* = 9–16, *N_p_* = 3500 for *n* = 17–40, *N_p_* = 2500 for *n* = 41–60, *N_p_* = 1500 for *n* = 61–80, and *N_p_* = 1000 for *n* = 81–160. For 2BrPyr, we used an additional set of N_p_ = 500 points for *n* = 161–170. This represents a total of 701,000 computed excitation energies and oscillator strengths.

While the NEA is able to describe the overall shapes and intensities of the absorption bands, it cannot describe vibrational progressions, which would require treating the nuclei quantum-mechanically. As discussed in Section 2.1, Band I of both bromopyrimidines presents very rich vibrational structures, whose interpretation becomes impractical without support from theory. Given the limitations of the NEA calculations, we have also employed a second theoretical approach, based on FCHT analysis, as performed with the time-independent procedure [63,64,65]. The excited-state potential energy surface is described with the adiabatic Hessian model, where force constants and vibrational frequencies are computed at the equilibrium geometry of the excited state. Ground state anharmonic frequencies were computed with the generalized second-order vibrational perturbation method [64,66], and from those we also obtained excited state anharmonic frequencies by employing a mode-specific scaling factor built from the Duschinsky matrix [67]. Temperature effects are accounted for by considering excitations from vibrationally excited levels with populations larger than 2%, for a temperature of 298 K, corresponding to the present experimental condition. All the calculations required for the FCHT analysis were performed with the Gaussian 16 software [59].

## 4. Conclusions

Absolute photoabsorption cross sections obtained with high resolution VUV synchrotron radiation in the 3.7–10.8 eV energy range are reported for 2BrPyr and 5BrPyr. Theoretical cross sections are also presented, as computed within the NEA in combination with TDDFT calculations. The good agreement between measurements and calculations gives strong support for the interpretation and discussion of both obtained VUV spectra.

The present study, together with a previous report on the photoabsorption of fluoranil [50] showed that the NEA can be successfully employed to probe photoabsorption for considerably larger energies, closer to the ionization threshold. While the improved accuracy of the NEA with respect to the simpler vertical approximation is well known for the low energy regime, it remained to be demonstrated in practice for higher energies. However, the overall computational cost increases sharply when the NEA is employed for higher energies, requiring the evaluation of dozens or hundreds of excited states for an extensive set of geometries.

In conclusion, the lowest lying band (3.7–4.6 eV) presents the lowest cross sections, and it was assigned to the first singlet excited state, a π*(a_2_) ← n_-_(b_2_) transition. With the support of FCHT calculations, we placed the band origin of 2BrPyr at 3.87 eV and of 5BrPyr at 3.79 eV, whereas a number of low intensity hot bands and main vibrational progressions could be assigned to C-Br rocking/wagging modes and to totally symmetric ring stretching modes found with frequencies in the 700~1200 cm^−1^ range, respectively. The Band II (4.6–5.4 eV) was attributed to a π*(a_2_) ← π(b_1_) transition, which is followed by a third band (5.4–6.5 eV) and assigned to a π*(b_1_) ← π(b_1_) transition, the latter displaying no vibrational structures. For the three lowest-lying bands, the cross sections of both 2BrPyr and 5BrPyr are rather similar, the first two being slightly red-shifted in 5BrPyr, and the third being somewhat more intense in 2BrPyr. Still in Band III, a weak shoulder was observed for 5BrPyr, and explained by a larger effect of the bromine atom position on the σ*_Br_(a_1_) ← π_r_(b_1_) transition.

Going up in energy we have the most intense absorption bands, IV (6.5–7.3 eV) and V (7.3–9.0 eV), which should be analyzed together. As a whole, their large intensities stem from the four possible excitations from the π_Br_(b_1_) and π(a_2_) occupied orbitals, to the π*(b_1_) and π*(a_2_) unoccupied orbitals. The analogous excitations account for the main absorption bands of the related bromobenzene, pyrimidine, and benzene molecules. Compared with bromobenzene, the band of both bromopyrimidines is displaced to higher energies and broadens significantly, such that some individual π* ← π transitions become apparent. Even so, the isomer effect is more substantial in this energy region, affecting the behavior of the individual π* ← π excitations and of other, less intense transitions. Finally, Band VI (9.0–10.8 eV) should involve many more valence and Rydberg excited states, but despite this, broader features could still be interpreted. In this regime, both spectra showed distinct vibrational progressions.

An extensive comparison between the VUV spectra of bromopyrimidines and the available data for bromobenzene revealed a series of similarities and differences. Namely, for the bromopyrimidines we observed a new band (Band I), broader (Band II) or a complete suppression of vibrational structures (Band III), broadened and blue shifted features (Bands IV and V), and a loss of intensity (Bands II and VI). An additional key distinction concerns the Rydberg states, much less pronounced in both bromopyrimidines than in bromobenzene. A similar trend can be noticed when comparing the spectra of pyrimidine and benzene.

This work is the most comprehensive VUV photoabsorption study yet reported for 2BrPyr and 5BrPyr. Considering that bromopyrimidines are known for their potential as radiosensitizers, the detailed picture we have provided on their excited states and VUV photoabsorption spectra could bring important insights on the production of reactive radicals and on radiation damage in general.

## Figures and Tables

**Table 1:** Calculated vertical excitation energies and vertical oscillator strengths (f_0_) of 2-bromopyrimidine, computed at the CAM-B3LYP/aug-cc-pVDZ+2s2p2d level of theory, compared with the experimental data. In the main character column, we present the dominant hole and particle natural transition orbitals and their corresponding occupation numbers.

2-bromopyrimidine						
Experimental			Theoretical			
Band	Energy (eV)	Cross Section (Mb)	State	Energy (eV)	f_0_	Main Character
I	4.25	0.9	1B_1_	4.55	0.0037	0.99 n_-_(b_2_) π*(a_2_)
II	4.85	6.5	1A_2_	4.98	0	0.99 n_-_(b_2_) π*(b_1_)
			1B_2_	5.40	0.0343	0.89 π(b_1_) π*(a_2_)
III	5.91	36.9	1A_1_	6.03	0.2446	0.95 π(b_1_) π*(b_1_)
			2A_2_	6.15	0	0.97 n_+_(a_1_) π*(a_2_)
			2B_1_	6.34	0.0014	0.86 π(b_1_) σ*_Br_(a_1_)
			3A_2_	6.34	0	0.99 n_Br_(b_2_) π*(b_1_)
			3B_1_	6.42	0.0006	0.87 n_Br_(b_2_) π*(a_2_)
			2B_2_	6.45	0.0014	0.98 n_Br_(b_2_) σ*_Br_(a_1_)
IV	7.09	60.2	4B_1_	6.53	0.0050	0.96 n_+_(a_1_) π*(b_1_)
			3B_2_	6.97	0.0001	0.87 n_-_(b_2_) 3s(a_1_)
			5B_1_	7.00	0.0450	0.97 π(b_1_) 3s(a_1_)
			4B_2_	7.02	0.0014	0.71 π_Br_(b_1_) π*(a_2_) + 0.28 π(a_2_) π*(b_1_)
			2A_1_	7.24	0.1241	0.60 π(a_2_) π*(a_2_) + 0.33 π_Br_(b_1_) π*(b_1_)
			5B_2_	7.33	0.0002	0.88 n_-_(b_2_) σ*_Br_(a_1_)
			6B_1_	7.50	0.0114	0.92 π(b_1_) 3p_z_(a_1_)
V	8.04	99.9	6B_2_	7.56	0.0000	0.74 n_Br_/n_-_(b_2_) 3p_z_(a_1_) + 0.24 n_Br_/n_-_(b_2_) 3s/σ*_Br_(a_1_)
			3A_1_	7.62	0.0045	0.88 n_-_(b_2_) 3p_y_(b_2_)
			4A_1_	7.75	0.1902	0.61 π(b_1_) 3p_x_(b_1_) + 0.23 π_Br_(b_1_) π*(b_1_)
			4A_2_	7.75	0	0.96 π(b_1_) 3p_y_(b_2_)
			5A_2_	7.80	0	0.95 n_-_(b_2_) 3p_x_(b_1_)
			5A_1_	7.87	0.2166	0.45 π(b_1_) 3p_x_(b_1_) + 0.30 π_Br_(b_1_) π*(b_1_)
			7B_2_	7.91	0.0098	0.63 n_-_(b_2_) 4s(a_1_) + 0.33 n_Br_(b_2_) 3s(a_1_)
			8B_2_	8.00	0.4315	0.51 π(a_2_) π*(b_1_) + 0.33 π_Br_(b_1_) π*(a_2_)
			7B_1_	8.07	0.0001	0.95 π(b_1_) 3d_x2_/3d_z2_(a_1_)
			9B_2_	8.18	0.0192	0.85 n_-_(b_2_) 3d_x2-y2_(a_1_)
			6A_2_	8.24	0	0.95 π(b_1_) 3d_yz_(b_2_)
			6A_1_	8.29	0.0098	0.71 n_-_/n_Br_(b_2_) 3d_yz_(b_2_) + 0.19 π(b_1_) 3d_xz_(b_1_)
			8B_1_	8.30	0.0163	0.96 π(b_1_) 3d_y2_(a_1_)
			10B_2_	8.31	0.0004	0.68 n_Br_/n_-_(b_2_) σ*_Br_/3d_z2_(a_1_)
			7A_1_	8.32	0.0093	0.73 π(b_1_) 3d_xz_(b_1_) + 0.17 n_-_/n_Br_(b_2_) 3d_yz_(b_2_)
			7A_2_	8.36	0	0.81 n_Br_/n_-_(b_2_) 3d_xz_(b_1_)
			9B_1_	8.36	0.0003	0.93 π(b_1_) 3d_y2_(a_1_)
			11B_2_	8.40	0.0112	0.93 n_-_/n_Br_(b_2_) 4s/σ*_Br_(a_1_)
			10B_1_	8.42	0.0002	0.96 n_-_/n_Br_(b_2_) 3d_xy_(a_2_)
			12B_2_	8.44	0.0021	0.99 π(b_1_) 3d_xy_(a_2_)
			8A_1_	8.45	0.0001	0.49 n_+_(a_1_) σ*_Br_/4s(a_1_) + 0.34 n_Br_/n_-_(b_2_) 3p_y_(b_2_)
			11B_1_	8.48	0.0014	0.95 π(b_1_) 4s(a_1_)
			9A_1_	8.52	0.0050	0.87 π(b_1_) 4p_x_(b_1_)
			8A_2_	8.53	0	0.96 π(b_1_) 4p_y_(b_2_)
			11A_1_	8.69	0.0700	0.38 n_+_(a_1_) σ*_Br_(a_1_) + 0.37 n_Br_(b_2_) 3p_y_(b_2_)
			12A_1_	8.75	0.0818	0.64 n_+_(a_1_) σ*_Br_(a_1_) + 0.30 n_Br_(b_2_) 3p_y_(b_2_)
			20B_1_	9.34	0.0319	0.75 π(a_2_) σ*_CH_/3p_y_(b_2_)
			22A_1_	9.92	0.0603	0.65 n_+_(a_1_) 3d_yz_(a_1_) + 0.25 n_Br_(b_2_) 3d_y2-z2_(b_2_)

**Table 2:** Calculated vertical excitation energies and vertical oscillator strengths (f_0_) of 5-bromopyrimidine, computed at the LYP/aug-cc-pVDZ+2s2p2d level of theory, compared with the experimental data. In the main character column, we present the dominant hole and particle natural transition orbitals and their corresponding occupation numbers.

5-bromopyrimidine						
Experimental			Theoretical			
Band	Energy (eV)	Cross Section (Mb)	State	Energy (eV)	f_0_	Main Character
I	4.21	1.2	1B_1_	4.48	0.0059	0.99 n_-_(b_2_) π*(a_2_)
II	4.80	6.3	1A_2_	4.89	0	0.99 n_-_(b_2_) π*(b_1_)
			1B_2_	5.35	0.0349	0.89 π_r_(b_1_) π*(a_2_)
III	5.98	26.0	2B_1_	5.89	0.0002	0.99 π(b_1_) σ*_Br_(a_1_)
			2A_2_	6.03	0	0.97 n_+_(a_1_) π*(a_2_)
			1A_1_	6.07	0.2129	0.94 π(b_1_) π*(b_1_)
			3B_1_	6.39	0.0049	0.99 n_+_(a_1_) π*(b_1_)
			2B_2_	6.57	0.0003	0.95 n_Br_(b_2_) σ*_Br_(a_1_)
IV	7.00	45.6	4B_1_	6.86	0.0020	0.98 n_Br_(b_2_) π*(a_2_)
			5B_1_	6.89	0.0162	0.97 π(b_1_) 3s(a_1_)
			3B_2_	6.98	0.0032	0.74 n_-_(b_2_) 3s/σ*_Br_(a_1_) + 0.24 n_Br_(b_2_) σ*_Br_(a_1_)
			3A_2_	7.10	0	0.99 n_Br_(b_2_) π*(b_1_)
			4B_2_	7.23	0.0474	0.51 π(a_2_) π*(b_1_) + 0.46 π(b_1_) π*(a_2_)
			2A_1_	7.34	0.2217	0.78 π(a_2_) π*(a_2_) + 0.10 π(b_1_) π*(b_1_)
V	8.10	110.8	5B_2_	7.36	0.0002	0.83 n_-_(b_2_) σ*_Br_(a_1_)
			3A_1_	7.63	0.0273	0.73 n_-_(b_2_) 3p_y_(b_2_) + 0.14 n_+_(a_1_) σ*_Br_/3s(a_1_)
			4A_2_	7.66	0	0.99 π(b_1_) 3p_y_(b_2_)
			6B_2_	7.67	0.0686	0.88 n_-_(b_2_) 3p_z_(a_1_)
			6B_1_	7.69	0.0028	0.96 π(b_1_) 3p_z_(a_1_)
			4A_1_	7.69	0.0060	0.88 π(b_1_) 3p_x_(b_1_)
			5A_2_	7.73	0	0.97 n_-_(b_2_) 3p_x_(b_1_)
			7B_2_	7.99	0.0056	0.54 n_Br_/n_-_(b_2_) 3s(a_1_) + 0.25 n_Br_/n_-_(b_2_) 3d_x2-z2_(a_1_)
			7B_1_	8.06	0.0000	0.96 π(b_1_) 3d_x2_(a_1_)
			8B_2_	8.13	0.0107	0.82 n_-_(b_2_) 3d_x2_(a_1_)
			9B_2_	8.18	0.3188	0.43 π_Br_(b_1_) π*(a_2_) + 0.25 π(a_2_) π*(b_1_)
			8B_1_	8.21	0.0014	0.81 π_Br_(b_1_) σ*_Br_(a_1_)
			5A_1_	8.28	0.1517	0.65 π_Br_(b_1_) π*(b_1_) + 0.25 π(b_1_) 3d_xz_(b_1_)
			9B_1_	8.29	0.0063	0.88 π(b_1_) 3d_y2-z2_(a_1_)
			6A_1_	8.31	0.0218	0.33 n_-_(b_2_) 3d_yz_(b_2_) + 0.33 π(b_1_) 3d_xz_(b_1_)
			10B_1_	8.36	0.0081	0.98 π(b_1_) 3d_z2_(a_1_)
			6A_2_	8.36	0	0.97 π(b_1_) 3d_yz_(b_2_)
			7A_2_	8.38	0	0.96 π(a_2_) σ*_Br_(a_1_)
			7A_1_	8.38	0.0430	0.45 π(b_1_) 3d_xz_(b_1_) + 0.28 n_-_(b_2_)
			10B_2_	8.38	0.0249	0.93 π(b_1_) 3d_xy_(a_2_)
			11B_1_	8.42	0.0016	0.95 n_-_(b_2_) 3d_xy_(a_2_)
			11B_2_	8.45	0.0261	0.79 n_-_(b_2_) 3d_y2-z2_(a_1_) + 0.11 n_Br_(b_2_) 3s(a_1_)
			12B_2_	8.45	0.0001	0.99 n_-_(b_2_) 4s(a_1_)
			8A_2_	8.47	0	0.96 n_-_(b_2_) 3d_xz_(b_1_)
			8A_1_	8.48	0.0460	0.64 n_-_(b_2_) 3d_yz_(b_2_) + 0.27 n_+_(a_1_) σ*_Br_/3s(a_1_)
			9A_1_	8.51	0.0021	0.92 π(b_1_) 4p_x_(b_1_)
			11A_1_	8.81	0.2800	0.62 n_+_(a_1_) σ*_Br_(a_1_) + 0.31 n_Br_(b_2_) 3p_y_(b_2_)
			12A_1_	8.92	0.0683	0.45 n_Br_(b_2_) 3p_y_(b_2_) + 0.37 n_+_(a_1_) σ*_Br_(a_1_)

**Table 3:** Proposed vibrational assignments in the 4.6−5.4 eV absorption band of 2BrPyr (Band II). Δ*E* (in eV) represents the energy of one extra quanta in the vibrational progression. The symbol ‘‘/’’ means both modes are involved.

Energy (eV)	Assignment	$\Delta E\;\left(\upsilon_{7}^{\prime }\right)$	$\Delta E\;\left(\upsilon_{24}^{\prime }\right)$
4.73	$0_{0}^{0}$		
4.85	$7_{0}^{1}$	0.12	
4.88 (b)	$24_{0}^{1}/7_{0}^{1}$	0.12	0.030
5.97 (b)	$7_{0}^{2}$	0.12	
5.00 (s)	$24_{0}^{1}/7_{0}^{2}$	0.12	0.029
5.09 (b)	$7_{0}^{3}$	0.12	
5.12 (b)	$24_{0}^{1}/7_{0}^{3}$	0.12	0.029
5.24 (b)	$24_{0}^{1}/7_{0}^{4}$	0.12	
4.73	$0_{0}^{0}$		
4.85	$7_{0}^{1}$	0.12	
4.88 (b)	$24_{0}^{1}/7_{0}^{1}$	0.12	0.030
5.97 (b)	$7_{0}^{2}$	0.12	

(b) broad structure; (s) shoulder structure.

**Table 4:** Proposed vibrational assignments in the 4.6−5.4 eV absorption band of 5BrPyr (Band II). Δ*E* (in eV) represents the energy of one extra quanta in the vibrational progression. The symbol ‘‘/’’ means both modes are involved.

Energy (eV)	Assignment	$\Delta E\;\left(\upsilon_{7}^{\prime }\right)$	$\Delta E\;\left(\upsilon_{24}^{\prime }\right)$
4.68	$0_{0}^{0}$		
4.70 (b)	$24_{0}^{1}$		0.03
4.80	$7_{0}^{1}$	0.13	
4.83 (b)	$24_{0}^{2}/7_{0}^{1}$	0.12	0.03
4.92	$7_{0}^{2}$	0.12	
4.95 (b)	$24_{0}^{3}/7_{0}^{2}$	0.13	0.03
5.05	$7_{0}^{3}$	0.12	
5.07 (b)	$24_{0}^{4}/7_{0}^{3}$	0.12	0.03
5.17 (b)	$7_{0}^{4}$	0.13	
5.20 (b)	$24_{0}^{5}/7_{0}^{4}$	0.13	0.03

(b) broad structure.

**Table 5:** Proposed vibrational assignments in the 7.3−9.0 eV absorption band of 5BrPyr (Band V). Δ*E* (in eV) represents the energy of one extra quanta in the vibrational progression. The symbol ‘‘/’’ means both modes are involved; the symbol ‘‘;’’ means two alternative assignments.

Energy (eV)	Assignment	$\Delta E\;\left(\upsilon_{4}^{\prime };\upsilon_{19}^{\prime }\right)$	$\Delta E\;\left(\upsilon_{9}^{\prime }\right)$	$\Delta E\;\left(\upsilon_{23}^{\prime }\right)$
7.38	$0_{0}^{0}$			
7.41	$9_{0}^{1}$		0.03	
7.46	$23_{0}^{1}$			0.09
7.50	$9_{0}^{2}/23_{0}^{1}$		0.03	0.09
7.55	$23_{0}^{2}/4_{0}^{1};19_{0}^{1}$	0.170		0.09
7.59	$9_{0}^{3}/23_{0}^{2}$		0.04	0.09
7.63	$23_{0}^{3}$			0.08
7.68	$9_{0}^{4}/23_{0}^{3}$		0.03	0.09
7.72	$23_{0}^{4}/4_{0}^{2};19_{0}^{2}$	0.170		0.09
7.76 (s)	$9_{0}^{5}/23_{0}^{4}$		0.04	0.09
7.90 (s)	$4_{0}^{3};19_{0}^{3}$	0.180		
7.99 (s)	$0_{0}^{0}$			
8.03	$9_{0}^{1}$		0.04	
8.06	$9_{0}^{2}/23_{0}^{1}/4_{0}^{4};19_{0}^{4}$	0.160	0.04	0.07
8.10	$9_{0}^{3}/23_{0}^{1}$		0.04	0.07
8.14 (w)	$9_{0}^{4}/23_{0}^{2}$		0.04	0.08
8.17	$9_{0}^{5}/23_{0}^{2}$		0.03	0.08
8.22	$9_{0}^{6}/23_{0}^{3}$		0.04	0.08
8.25	$9_{0}^{7}/23_{0}^{3}/4_{0}^{5};19_{0}^{5}$	0.190	0.04	0.08
8.29	$9_{0}^{8}/23_{0}^{4}$		0.05	0.07
8.32	$9_{0}^{9}/23_{0}^{4}$		0.04	0.07
8.36	$9_{0}^{10}/23_{0}^{5}$		0.04	0.07
8.39	$9_{0}^{11}/23_{0}^{5}$		0.04	0.07
8.43 (s)	$9_{0}^{12}/23_{0}^{6}/4_{0}^{6};19_{0}^{6}$	0.170	0.04	0.07
8.47 (s)	$9_{0}^{13}/23_{0}^{6}$		0.04	0.07
8.53	$0_{0}^{0}$			
8.57	$9_{0}^{1}$		0.04	
8.60	$9_{0}^{2}/23_{0}^{1}/4_{0}^{7};19_{0}^{7}$	0.190	0.04	0.08
8.65	$9_{0}^{3}/23_{0}^{1}$		0.05	0.08
8.70	$9_{0}^{4}/23_{0}^{2}$		0.05	0.09
8.73	$9_{0}^{5}/23_{0}^{2}$		0.04	0.09
8.77	$9_{0}^{6}/23_{0}^{3}$		0.04	0.08
8.82	$9_{0}^{7}/23_{0}^{3}/4_{0}^{8};19_{0}^{8}$	0.210	0.05	0.08
8.85	$9_{0}^{8}/23_{0}^{4}$		0.04	0.08
8.89	$9_{0}^{9}/23_{0}^{4}$		0.04	0.07
8.94	$9_{0}^{10}/23_{0}^{5}$		0.05	0.09
8.97	$9_{0}^{11}/23_{0}^{5}$		0.03	0.08

(s) shoulder structure; (w) weak feature.

**Table 6:** Proposed vibrational assignments in the 7.3−9.0 eV absorption band of 2BrPyr (Band V). Δ*E* (in eV) represents the energy of one extra quanta in the vibrational progression. The symbol ‘‘/’’ means both modes are involved; the symbol ‘‘;’’ means two alternative assignments.

Energy (eV)	Assignment	$\Delta E\;\left(\upsilon_{9}^{\prime };\upsilon_{24}^{\prime }\right)$	$\Delta E\;\left(\upsilon_{23}^{\prime }\right)$	$\Delta E\;\left(\upsilon_{8}^{\prime }\right)$	$\Delta E\;\left(\upsilon_{5}^{\prime };\upsilon_{6}^{\prime };\upsilon_{7}^{\prime }\right)$
7.6	$0_{0}^{0}$				
7.64 (s)	$9_{0}^{1};24_{0}^{1}$	0.040			
7.67	$9_{0}^{2};24_{0}^{2}$	0.035			
7.71	$9_{0}^{3};24_{0}^{3}$	0.034			
7.74	$9_{0}^{4};24_{0}^{4}$/$5_{0}^{4}$; $6_{0}^{4}$; $7_{0}^{4}$	0.034			0.143
7.78 (w)	$9_{0}^{5};24_{0}^{5}$	0.034			
7.81 (w)	$9_{0}^{6};24_{0}^{6}$	0.034			
7.84 (w)	$9_{0}^{7};24_{0}^{7}$	0.034			
7.88 (w)	$9_{0}^{8};24_{0}^{8}$/$5_{0}^{4}$; $6_{0}^{4}$; $7_{0}^{4}$	0.036			0.138
7.92 (w)	$9_{0}^{9};24_{0}^{9}$	0.040			
8.04	$0_{0}^{0}$				
8.11 (s)	$23_{0}^{1}$		0.076		
8.19 (s)	$23_{0}^{2}$		0.076		
8.30	$23_{0}^{3}$		0.080		
8.40	$0_{0}^{0}$				
8.50 (s)	$8_{0}^{1}$			0.095	
8.59 (s)	$8_{0}^{2}$			0.095	
8.70 (b)	$8_{0}^{3}$			0.095	
8.79 (b)	$8_{0}^{4}$			0.095	

(b) broad structure; (s) shoulder structure; (w) weak feature.

**Table 7:** Proposed vibrational assignments in the 9.0−10.8 eV absorption band of 5BrPyr (Band VI). Δ*E* (in eV) represents the energy of one extra quanta in the vibrational progression. The symbol ‘‘/’’ means both modes are involved; the symbol ‘‘;’’ means two alternative assignments.

Energy (eV)	Assignment	$\Delta E\;\left(\upsilon_{9}^{\prime }\right)$	$\Delta E\;\left(\upsilon_{23}^{\prime }\right)$	$\Delta E\;\left(\upsilon_{24}^{\prime }\right)$
9.06	$0_{0}^{0}$			
9.09	$24_{0}^{1}$			0.03
9.11	$24_{0}^{2}$			0.02
9.14	$23_{0}^{1}$		0.08	
9.17	$23_{0}^{1}/24_{0}^{1}$		0.08	0.03
9.21	$23_{0}^{1}/24_{0}^{2}$		0.10	0.04
9.23	$23_{0}^{2}$		0.09	
9.26	$23_{0}^{2}/24_{0}^{1}$		0.09	0.03
9.29 (s)	$23_{0}^{2}/24_{0}^{2}$		0.08	0.03
9.32 (s)	$23_{0}^{3}$		0.09	
9.34	$23_{0}^{3}/24_{0}^{1}$		0.08	0.02
9.37	$23_{0}^{3}/24_{0}^{2}$		0.08	0.03
9.41	$23_{0}^{4}$		0.09	
9.43	$23_{0}^{4}/24_{0}^{1}$		0.09	0.02
9.45	$23_{0}^{4}/24_{0}^{2}$		0.08	0.02
9.49	$23_{0}^{5}$		0.08	
9.52	$23_{0}^{5}/24_{0}^{1}$		0.09	0.03
9.54	$23_{0}^{5}/24_{0}^{2}$		0.09	0.02
9.57	$23_{0}^{6}$		0.08	
9.60	$23_{0}^{6}/24_{0}^{1}$		0.08	0.03
9.63 (s)	$23_{0}^{6}/24_{0}^{2}$		0.09	0.03
9.66	$23_{0}^{7}$		0.09	
9.69	$23_{0}^{7}/24_{0}^{1}$		0.09	0.03
9.72 (w)	$23_{0}^{7}/24_{0}^{2}$		0.09	0.03
9.75	$23_{0}^{8}$		0.09	
9.79	$23_{0}^{8}/24_{0}^{1}$		0.10	0.04
9.83	$23_{0}^{9}$		0.08	
9.86 (s) (b)	$23_{0}^{9}/24_{0}^{1}$		0.07	0.03
9.90 (b)	$23_{0}^{10}$		0.07	
9.95 (b)	$23_{0}^{10}/24_{0}^{1}$		0.07	0.05
10.00 (w)	$23_{0}^{11}$		0.10	
10.02	$23_{0}^{11}/24_{0}^{1}$		0.09	0.02
10.07	$23_{0}^{12}$		0.07	
10.10	$23_{0}^{12}/24_{0}^{1}$		0.07	0.03
10.15	$23_{0}^{13}$		0.08	
10.18	$23_{0}^{13}/24_{0}^{1}$		0.08	0.03
10.24 (w)	$23_{0}^{14}$		0.09	
10.27	$23_{0}^{14}/24_{0}^{1}$		0.09	0.03
10.32 (w)	$23_{0}^{15}$		0.08	
10.35 (w)	$23_{0}^{15}/24_{0}^{1}$		0.08	0.03
10.41				
10.45 (s)	$9_{0}^{1}$	0.04		
10.49	$23_{0}^{1}/9_{0}^{2}$	0.04	0.08	
10.54	$9_{0}^{3}$	0.05		
10.57	$23_{0}^{2}/9_{0}^{4}$	0.05	0.08	
10.61 (w)	$9_{0}^{5}$	0.04		
10.67	$23_{0}^{3}/9_{0}^{6}$	0.06	0.09	
10.71	$9_{0}^{7}$	0.05		
10.75	$23_{0}^{4}/9_{0}^{8}$	0.06	0.07	

(b) broad structure; (s) shoulder structure; (w) weak feature.

**Table 8:** Proposed vibrational assignments in the 9.0−10.8 eV absorption band of 2BrPyr (Band VI). Δ*E* (in eV) represents the energy of one extra quanta in the vibrational progression. The symbol ‘‘/’’ means both modes are involved; the symbol ‘‘;’’ means two alternative assignments.

Energy (eV)	Assignment	$\Delta E\;\left(\upsilon_{9}^{\prime };\upsilon_{24}^{\prime }\right)$	$\Delta E\;\left(\upsilon_{7}^{\prime }\right)$	$\Delta E\;\left(\upsilon_{5}^{\prime };\upsilon_{6}^{\prime };\upsilon_{7}^{\prime }\right)$
9.07	$0_{0}^{0}$			
9.10 (w)	$9_{0}^{1};24_{0}^{1}$	0.033		
9.14 (w)	$9_{0}^{2};24_{0}^{2}$	0.040		
9.17	$9_{0}^{3};24_{0}^{3}/7_{0}^{1}$	0.034	0.112	
9.21	$9_{0}^{4};24_{0}^{4}$	0.036		
9.24	$9_{0}^{5};24_{0}^{5}$	0.040		
9.28	$9_{0}^{6};24_{0}^{6}/7_{0}^{2}$	0.042	0.110	
9.32	$9_{0}^{7};24_{0}^{7}$	0.042		
9.36	$9_{0}^{8};24_{0}^{8}$	0.042		
9.41	$9_{0}^{9};24_{0}^{9}/7_{0}^{3}$	0.037	0.127	
9.45	$9_{0}^{10};24_{0}^{10}$	0.033		
9.48 (w)	$9_{0}^{11};24_{0}^{11}$	0.035		
9.51	$9_{0}^{12};24_{0}^{12}$	0.034		
9.55 (w)	$9_{0}^{13};24_{0}^{13}$	0.035		
9.58 (s)	$9_{0}^{14};24_{0}^{14}$	0.035		
9.61	$9_{0}^{15};24_{0}^{15}$	0.035		
9.65	$9_{0}^{16};24_{0}^{16}$	0.035		
9.69 (s)	$9_{0}^{17};24_{0}^{17}$	0.035		
9.73 (s)	$9_{0}^{18};24_{0}^{18}$	0.035		
9.77	$5_{0}^{1};6_{0}^{1};7_{0}^{1}$			0.150
9.93 (s)	$5_{0}^{2};6_{0}^{2};7_{0}^{2}$			0.150
10.08	$5_{0}^{3};6_{0}^{3};7_{0}^{3}$			0.158
10.23 (s)	$5_{0}^{4};6_{0}^{4};7_{0}^{4}$			0.142
10.36	$5_{0}^{5};6_{0}^{5};7_{0}^{5}$			0.137
10.52 (b)	$5_{0}^{6};6_{0}^{6};7_{0}^{6}$			0.154
10.65	$5_{0}^{7};6_{0}^{7};7_{0}^{7}$			0.139

(b) broad structure; (s) shoulder structure; (w) weak feature.

## Data Availability

The data presented in this study are available on request from the corresponding author.

## Supplementary Materials

The following are available online at https://www.mdpi.com/article/10.3390/ijms22126460/s1.
