# Supplementary material for: Excited States of Bromopyrimidines Probed by VUV Photoabsorption Spectroscopy and Theoretical Calculations
Source: Int J Mol Sci. 2021 Jun 16;22(12):6460. doi: 10.3390/ijms22126460 (PMC8235550; doi:10.3390/ijms22126460)
Supplement: Supplementary file 1 [file ijms-22-06460-s001.zip › ijms-1248955-supplementary.pdf]

Supplementary Material

Excited States of Bromopyrimidines Probed by VUV Photoabsorption Spectroscopy and Theoretical Calculations

Mónica Mendes [1,*], Fábris Kossoski [2,*], Ana I. Lozano [1], João Pereira-da-Silva [1], Rodrigo Rodrigues [1], João Ameixa [1], Nykola C. Jones [3], Søren V. Hoffmann [3] and Filipe Ferreira da Silva [1]

[1] CEFITEC, Departamento de Física, NOVA School of Science and Technology, FCT NOVA, Universidade NOVA de Lisboa, 2829-516 Caparica, Portugal; ai.lozano@fct.unl.pt (A.I.L.); jvp.silva@campus.fct.unl.pt (J.P.-d.-S.); rar09843@campus.fct.unl.pt (R.R.); j.ameixa@campus.fct.unl.pt (J.A.); f.ferreiradasilva@fct.unl.pt (F.F.d.S.)

[2] Laboratoire de Chimie et Physique Quantiques (UMR 5626), Université de Toulouse, CNRS, UPS,
CEDEX 09, 31062 Toulouse, France

[3] ISA, Department of Physics and Astronomy, Aarhus University, Ny Munkegade 120, 8000 Aarhus C, Denmark; nykj@phys.au.dk (N.C.J.); vronning@phys.au.dk (S.V.H.)

* Correspondence: mf.mendes@fct.unl.pt (M.M.); fkossoski@irsamc.ups-tlse.fr (F.K.)

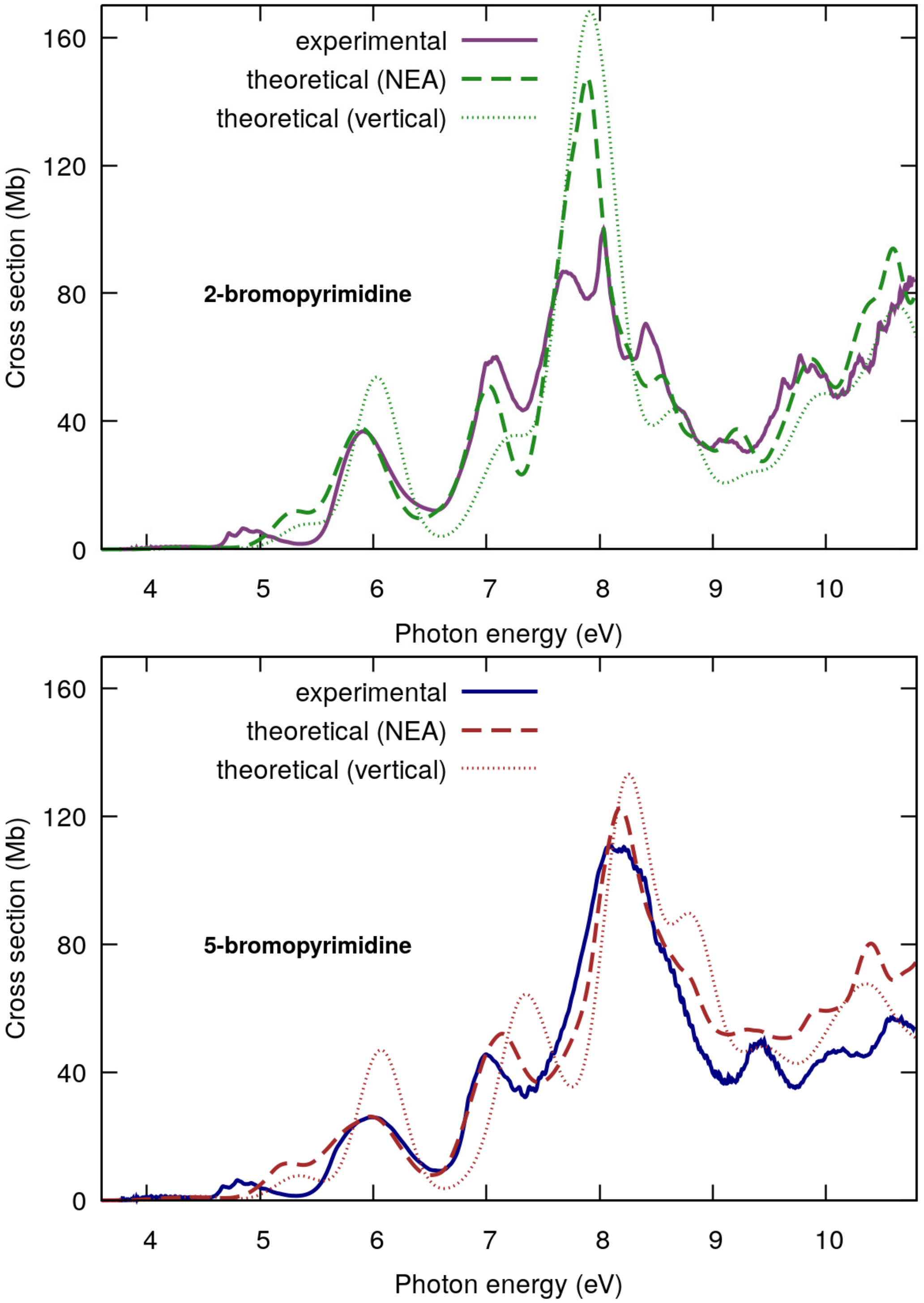

Figure S1. Comparison between measured and theoretical absorption cross sections for 2-bromopyrimidine (top) and 5-bromopyrimidine (bottom), the latter computed with the vertical approximation (for an arbitrary linewidth of 0.4 eV) and with the nuclear ensemble approach (NEA).

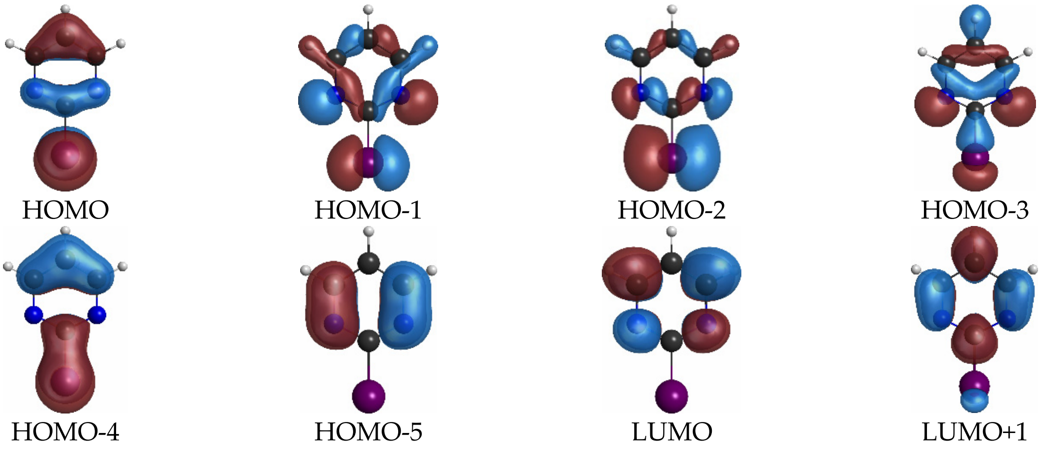

Figure S2. The frontier canonical orbitals for 2-bromopyrimidine.

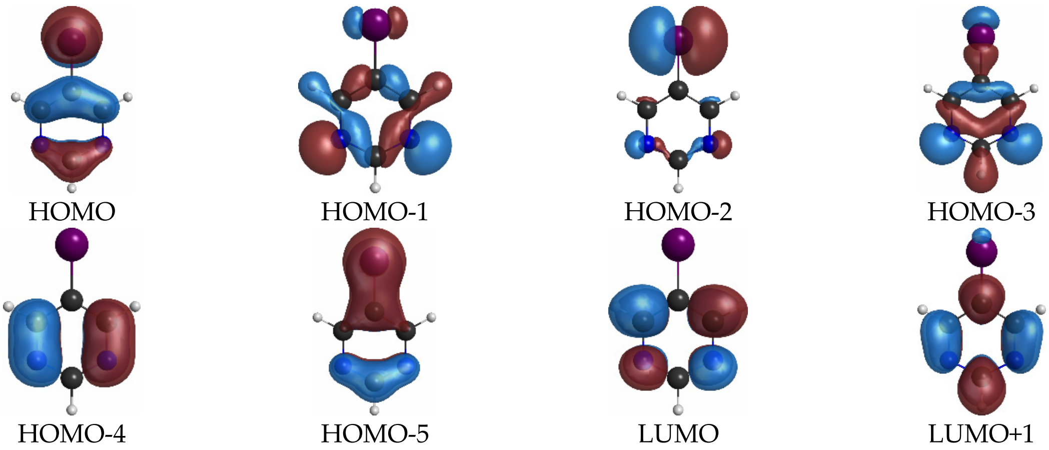

Figure S3. The frontier canonical orbitals for 5-bromopyrimidine.

Set of geometries optimized at the CAM-B3LYP/aug-cc-pVDZ+2s2p2d level of theory:

Ground state (S_0) geometry (in angstrom) of 2-bromopyrimidine

C	0.000000	0.000000	-0.144547
N	0.000000	1.189023	-0.724716
N	0.000000	-1.189023	-0.724716
C	0.000000	1.180041	-2.058947
C	0.000000	-1.180041	-2.058947
C	0.000000	0.000000	-2.791483
H	0.000000	2.155443	-2.548233
H	0.000000	-2.155443	-2.548233
H	0.000000	0.000000	-3.879105

Br 0.000000 0.000000 1.755575

First excited state (S_1) geometry (in angstrom) of 2-bromopyrimidine

C 0.000000 0.000000 -0.086024
N 0.000000 1.119786 -0.750749
N 0.000000 -1.119786 -0.750749
C 0.000000 1.192639 -2.129466
C 0.000000 -1.192639 -2.129466
C 0.000000 0.000000 -2.844207
H 0.000000 2.178314 -2.583072
H 0.000000 -2.178314 -2.583072
H 0.000000 0.000000 -3.933463
Br 0.000000 0.000000 1.792716

Ground state (S_0) geometry (in angstrom) of 5-bromopyrimidine

C 0.000000 0.000000 -2.764489
N 0.000000 1.189493 -2.166023
N 0.000000 -1.189493 -2.166023
C 0.000000 1.189164 -0.834710
C 0.000000 -1.189164 -0.834710
C 0.000000 0.000000 -0.115937
H 0.000000 2.155526 -0.328742
H 0.000000 -2.155526 -0.328742
Br 0.000000 0.000000 1.775297
H 0.000000 0.000000 -3.854496

First excited state (S_1) geometry (in angstrom) of 5-bromopyrimidine

C 0.196112 2.857635 0.000000
N 1.241155 2.085856 0.000000
N -0.997666 2.259002 0.000000
C 1.269757 0.721378 0.000000
C -1.124591 0.886310 0.000000
C 0.000000 0.099947 0.000000
H 2.219114 0.201108 0.000000
H -2.130184 0.475844 0.000000
Br -0.117817 -1.783517 0.000000
H 0.282577 3.940519 0.000000

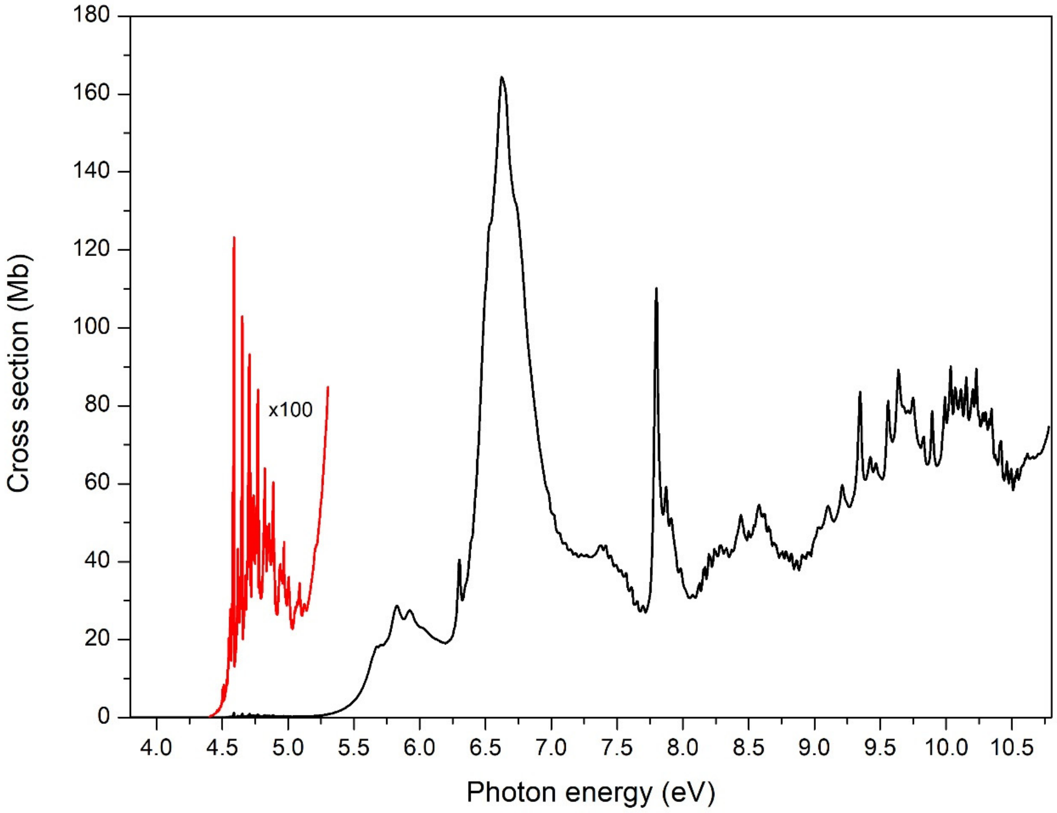

Figure S4. High-resolution VUV photoabsorption spectrum of bromobenzene in the 3.7–10.8 eV photon energy range. The original data was kindly provided by Palmer et al. [47] and can also be found online in the MPI-Mainz UV/VIS Spectral Atlas of Gaseous Molecules of Atmospheric Interest (www.uv-vis-spectral-atlas-mainz.org).

Table S1. Ground state (S_0) frequencies (in cm^{-1}) of 2-bromopyrimidine.

	Symmetry	Harmonic freq.	Anharmonic freq.	Character
1	a_1	3243.7	3114.9	H's stretching
2		3196.2	3065.5	H's stretching
3		1645.4	1606.5	ring stretching
4		1439.1	1413.1	ring stretching / H's symmetric rocking
5		1196.5	1178.0	ring stretching / H's symmetric rocking
6		1104.5	1090.0	ring stretching
7		1019.3	1005.9	ring stretching
8		756.9	748.9	ring stretching
9		336.7	336.6	C-Br stretching
10	a_2	1019.6	992.5	H's twisting
11		419.5	410.2	ring deformation
12	b_1	1024.3	998.4	H's wagging/twisting
13		823.8	810.5	H's wagging
14		803.8	787.5	ring deformation
15		486.3	476.1	ring deformation
16		150.9	150.9	Br wagging (out-of-plane)
17	b_2	3199.3	3083.5	H's stretching
18		1638.2	1596.1	ring stretching
19		1467.8	1442.8	ring stretching / H's symmetric rocking
20		1299.7	1282.4	H's asymmetric rocking
21		1244.1	1200.9	ring stretching
22		1115.4	1101.7	H rocking
23		637.6	633.0	ring deformation
24		274.5	273.4	Br rocking (in-plane)

Table S2. First excited state (S_0) frequencies (in cm^{-1}) of 2-bromopyrimidine.

	Symmetry	Harmonic freq.	Anharmonic freq.	Character
1	a_1	3267.9	3135.3	H's stretching
2		3225.8	3096.7	H's stretching
3		1530.7	1497.1	ring stretching
4		1439.3	1411.8	ring stretching / H's symmetric rocking
5		1163.9	1145.3	ring stretching / H's symmetric rocking
6		1071.6	1057.4	ring stretching
7		968.1	955.3	ring stretching
8		728.6	720.9	ring stretching
9		330.5	330.3	C-Br stretching
10	a_2	598.4	582.5	H's twisting
11		232.4	227.0	ring deformation
12	b_1	934.9	914.8	H wagging
13		709.3	695.2	H's wagging / ring deformation
14		549.1	538.0	ring deformation
15		481.3	471.5	ring deformation
16		168.9	168.8	Br wagging (out-of-plane)
17	b_2	3254.4	3136.7	H's stretching
18		1384.6	1350.8	ring stretching
19		1341.9	1311.6	ring stretching / H's symmetric rocking
20		1240.5	1217.2	H's asymmetric rocking
21		1035.4	1018.9	H rocking
22		873.2	856.9	ring stretching
23		370.8	367.6	ring deformation
24		194.1	193.0	Br rocking (in-plane)

Table S3. Ground state (S_0) frequencies (in cm^{-1}) of 5-bromopyrimidine.

	Symmetry	Harmonic freq.	Anharmonic freq.	Character
1	a_1	3212.0	3089.6	H’s stretching
2		3204.7	3060.7	H’s stretching
3		1640.0	1599.2	ring stretching
4		1458.6	1424.7	ring stretching / H’s symmetric rocking
5		1180.0	1168.3	H’s rocking / ring stretching
6		1128.7	1111.8	ring breathing
7		1045.6	1027.7	ring stretching
8		756.2	746.4	ring stretching
9		333.4	325.1	C-Br stretching
10	a_2	1006.3	975.4	H’s twisting / ring deformation
11		423.7	415.8	ring deformation
12	b_1	1034.6	1004.1	H’s wagging/twisting
13		948.4	921.4	H’s wagging / ring deformation
14		745.2	728.9	ring deformation
15		436.3	427.6	ring deformation
16		171.0	166.1	Br wagging (out-of-plane)
17	b_2	3205.0	3069.2	H’s stretching
18		1635.8	1597.2	ring stretching
19		1467.3	1440.6	H rocking / ring stretching
20		1365.3	1331.8	H’s symmetric rocking
21		1238.1	1208.7	ring stretching
22		1221.3	1198.5	ring stretching / H’s rocking
23		635.5	628.1	ring stretching
24		247.4	244.3	Br rocking (in-plane)

Table S4. First excited state (S_1) frequencies (in cm^{-1}) of 5-bromopyrimidine.

	Symmetry	Harmonic freq.	Anharmonic freq.	Character
1	a′	3287.1	3155.7	H stretching
2		3252.6	3109.6	H stretching
3		3246.2	3114.9	H stretching
4		1595.2	1557.2	ring stretching
5		1470.7	1436.9	ring stretching / H′s rocking
6		1382.3	1356.1	H rocking
7		1319.0	1289.3	ring stretching
8		1288.8	1257.5	H′s rocking
9		1165.6	1150.4	ring stretching / H′s rocking
10		1095.5	1078.4	ring stretching
11		1058.7	1039.8	ring stretching
12		989.4	972.6	ring stretching
13		726.1	713.0	ring stretching
14		715.3	705.1	ring stretching
15		327.5	319.6	C-Br stretching
16		251.2	248.1	Br rocking (in-plane)
17		225.7	222.3	ring stretching
18	a′′	933.7	906.8	H wagging
19		769.7	747.2	H wagging
20		574.5	560.4	ring deformation
21		513.1	501.5	ring deformation
22		447.8	436.0	H wagging
23		264.7	259.1	ring deformation
24		162.6	157.8	Br wagging (out-of-plane)

Table S5. Vibrational progression assignments for the low-energy tail (3.75 - 4.15 eV) of the absorption band I of 2-bromopyrimidine. From left to right, energies (in eV) of the experimental peak, and computed energies (in eV), intensities (in arbitrary units), and characters of the most important vibronic transitions between S_0 and S_1 electronic states. Several more transitions take place, however here we only show those with intensities larger than 0.5.

3.828	3.827	1.3	9^1 -> 0
	3.829	0.6	9^1;16^1 -> 16^1
3.845	3.846	0.7	11^1 -> 11^1
3.863	3.859	1.0	24^1 -> 24^1
3.868	3.868	5.7	0 -> 0
	3.871	2.6	16^1 -> 16^1
	3.873	1.2	16^2 -> 16^2
	3.875	0.5	16^3 -> 16^3
3.885	3.887	1.1	11^1 -> 9^1;11^1
3.902	3.899	1.6	24^1 -> 9^1;24^1
3.909	3.902	0.7	24^1;16^1 -> 9^1;24^1;16^1
	3.909	8.5	0 -> 9^1
	3.912	3.9	16^1 -> 9^1;16^1
	3.914	1.8	16^2 -> 9^1;16^2
	3.916	0.8	16^3 -> 9^1;16^3
	3.916	0.8	9^1 -> 8^1
	3.921	0.7	24^1 -> 23^1;9^1
3.924	3.928	0.8	11^1 -> 9^2;11^1
3.943	3.940	1.2	24^1 -> 9^2;24^1
	3.943	0.5	24^1;16^1 -> 9^2;24^1;16^1
3.949	3.945	1.0	9^1 -> 7^1
	3.948	0.6	24^1 -> 8^1;24^1
	3.950	1.2	9^1 -> 9^3
	3.950	6.3	0 -> 9^2
	3.952	0.6	9^1;16^1 -> 9^3;16^1
	3.953	2.9	16^1 -> 9^2;16^1

3.956	3.955	1.3	16^2 -> 9^2;16^2
	3.957	0.6	16^3 -> 9^2;16^3
	3.958	0.9	9^1 -> 6^1
	3.958	3.3	0 -> 8^1
	3.960	1.5	16^1 -> 8^1;16^1
	3.962	0.7	16^2 -> 8^1;16^2
	3.964	0.5	11^1 -> 7^1;11^1
3.965	3.969	1.4	9^1 -> 5^1
	3.971	0.6	9^1;16^1 -> 5^1;16^1
3.974	3.976	0.6	11^1 -> 8^1;9^1;11^1
	3.977	0.7	24^1 -> 7^1;24^1
	3.981	0.6	24^1 -> 9^3;24^1
3.988	3.987	4.1	0 -> 7^1
	3.988	0.7	11^1 -> 5^1;11^1
	3.989	0.9	24^1 -> 8^1;9^1;24^1
	3.989	1.9	16^1 -> 7^1;16^1
	3.990	0.7	24^1 -> 6^1;24^1
	3.991	1.1	9^1 -> 9^4
	3.991	3.1	0 -> 9^3
	3.991	0.9	16^2 -> 7^1;16^2
	3.993	0.5	9^1;16^1 -> 9^4;16^1
	3.994	1.4	16^1 -> 9^3;16^1
3.998	3.996	0.6	16^2 -> 9^3;16^2
	3.999	4.7	0 -> 8^1;9^1
	4.000	3.9	0 -> 6^1
	4.001	1.1	24^1 -> 5^1;24^1
	4.001	2.2	16^1 -> 8^1;9^1;16^1
	4.002	1.8	16^1 -> 6^1;16^1
	4.003	1.0	16^2 -> 8^1;9^1;16^2
	4.004	0.8	16^2 -> 6^1;16^2
	4.005	0.8	11^1 -> 7^1;9^1;11^1
4.006	4.010	5.8	0 -> 5^1
	4.013	2.7	16^1 -> 5^1;16^1
	4.015	1.2	16^2 -> 5^1;16^2
4.014	4.017	0.6	16^3 -> 5^1;16^3

	4.018	0.7	11^1 -> 6^1;9^1;11^1
	4.018	1.1	24^1 -> 7^1;9^1;24^1
	4.020	0.5	24^1;16^1 -> 7^1;9^1;24^1;16^1
4.024	4.028	6.1	0 -> 7^1;9^1
	4.029	1.1	11^1 -> 5^1;9^1;11^1
	4.030	0.6	24^1 -> 8^1;9^2;24^1
	4.030	2.8	16^1 -> 7^1;9^1;16^1
	4.031	1.0	24^1 -> 6^1;9^1;24^1
	4.032	0.6	9^1 -> 9^5
	4.032	1.1	0 -> 9^4
	4.032	1.3	16^2 -> 7^1;9^1;16^2
	4.035	0.5	16^1 -> 9^4;16^1
	4.035	0.6	16^3 -> 7^1;9^1;16^3
	4.035	0.6	9^1 -> 7^1;8^1
4.037	4.039	0.6	9^1 -> 8^1;9^3
	4.040	3.3	0 -> 8^1;9^2
	4.041	5.7	0 -> 6^1;9^1
	4.041	1.6	24^1 -> 5^1;9^1;24^1
	4.042	1.5	16^1 -> 8^1;9^2;16^1
	4.043	2.6	16^1 -> 6^1;9^1;16^1
	4.044	0.7	24^1;16^1 -> 5^1;9^1;24^1;16^1
	4.044	0.7	16^2 -> 8^1;9^2;16^2
	4.045	1.2	16^2 -> 6^1;9^1;16^2
	4.046	0.6	11^1 -> 7^1;9^2;11^1
4.045	4.047	0.6	16^3 -> 6^1;9^1;16^3
	4.047	0.6	9^1 -> 6^1;8^1
	4.047	0.9	0 -> 8^2
	4.051	8.5	0 -> 5^1;9^1
	4.054	3.9	16^1 -> 5^1;9^1;16^1
	4.054	1.0	0 -> 3^1
4.053	4.056	1.8	16^2 -> 5^1;9^1;16^2
	4.058	0.8	16^3 -> 5^1;9^1;16^3
	4.058	0.9	9^1 -> 5^1;8^1
	4.059	0.5	11^1 -> 6^1;9^2;11^1
	4.059	0.8	24^1 -> 7^1;9^2;24^1
	4.063	0.7	24^1 -> 5^1;23^1;9^1

4.063	4.068	0.8	9^1 -> 7^1;9^3
	4.069	4.5	0 -> 7^1;9^2
	4.070	0.8	11^1 -> 5^1;9^2;11^1
	4.071	2.1	16^1 -> 7^1;9^2;16^1
	4.072	0.8	24^1 -> 6^1;9^2;24^1
	4.073	0.9	16^2 -> 7^1;9^2;16^2
4.077	4.076	0.7	9^1 -> 6^1;7^1
	4.076	2.3	0 -> 7^1;8^1
	4.079	1.1	16^1 -> 7^1;8^1;16^1
	4.080	0.5	9^1 -> 8^1;9^4
	4.081	0.8	9^1 -> 6^1;9^3
	4.081	1.5	0 -> 8^1;9^3
	4.081	4.1	0 -> 6^1;9^2
	4.082	1.1	24^1 -> 5^1;9^2;24^1
	4.083	0.7	16^1 -> 8^1;9^3;16^1
	4.084	1.9	16^1 -> 6^1;9^2;16^1
	4.085	0.5	24^1;16^1 -> 5^1;9^2;24^1;16^1
	4.086	0.9	16^2 -> 6^1;9^2;16^2
4.085	4.087	1.0	9^1 -> 5^1;7^1
	4.088	1.2	0 -> 8^2;9^1
	4.089	2.2	0 -> 6^1;8^1
	4.090	0.6	24^1 -> 5^1;8^1;24^1
	4.090	0.6	16^1 -> 8^2;9^1;16^1
	4.091	1.0	16^1 -> 6^1;8^1;16^1
	4.092	1.1	9^1 -> 5^1;9^3
	4.092	6.2	0 -> 5^1;9^2
	4.094	0.5	9^1;16^1 -> 5^1;9^3;16^1
	4.095	2.8	16^1 -> 5^1;9^2;16^1
	4.095	1.5	0 -> 3^1;9^1
	4.097	1.3	16^2 -> 5^1;9^2;16^2
	4.097	0.7	16^1 -> 3^1;9^1;16^1
4.092	4.099	0.6	16^3 -> 5^1;9^2;16^3
	4.100	1.0	9^1 -> 5^1;6^1
	4.100	3.4	0 -> 5^1;8^1
	4.102	1.5	16^1 -> 5^1;8^1;16^1
	4.104	0.7	16^2 -> 5^1;8^1;16^2
4.102	4.105	1.4	0 -> 7^2

	4.106	0.5	11^1 -> 5^1;7^1;11^1
	4.107	0.6	24^1 -> 7^1;8^1;9^1;24^1
	4.108	0.6	16^1 -> 7^2;16^1
	4.108	0.5	24^1 -> 6^1;7^1;24^1
	4.109	0.8	9^1 -> 7^1;9^4
	4.110	2.1	0 -> 7^1;9^3
	4.111	0.7	9^1 -> 5^2
	4.112	1.0	16^1 -> 7^1;9^3;16^1
4.116	4.117	3.3	0 -> 7^1;8^1;9^1
	4.118	2.8	0 -> 6^1;7^1
	4.118	0.6	11^1 -> 5^1;8^1;9^1;11^1
	4.119	0.8	24^1 -> 5^1;7^1;24^1
	4.120	0.6	24^1 -> 6^1;8^1;9^1;24^1
	4.120	1.3	16^1 -> 6^1;7^1;16^1
	4.122	0.7	9^1 -> 6^1;9^4
	4.122	0.5	0 -> 8^1;9^4
	4.122	2.0	0 -> 6^1;9^3
	4.122	0.6	16^2 -> 6^1;7^1;16^2
	4.123	0.5	24^1 -> 5^1;9^3;24^1
	4.125	0.9	16^1 -> 6^1;9^3;16^1
4.125	4.129	4.3	0 -> 5^1;7^1
	4.129	0.8	0 -> 8^2;9^2
	4.130	3.1	0 -> 6^1;8^1;9^1
	4.131	1.3	0 -> 6^2
	4.131	0.9	24^1 -> 5^1;8^1;9^1;24^1
	4.131	2.0	16^1 -> 5^1;7^1;16^1
	4.132	0.7	24^1 -> 5^1;6^1;24^1
	4.132	1.4	16^1 -> 6^1;8^1;9^1;16^1
	4.133	1.0	9^1 -> 5^1;9^4
	4.133	0.6	16^1 -> 6^2;16^1
	4.133	2.9	0 -> 5^1;9^3
	4.133	0.9	16^2 -> 5^1;7^1;16^2
	4.134	0.7	16^2 -> 6^1;8^1;9^1;16^2
	4.136	1.3	16^1 -> 5^1;9^3;16^1
	4.136	1.1	0 -> 3^1;9^2
	4.136	0.5	11^1 -> 6^1;7^1;9^1;11^1
4.135	4.138	0.6	16^2 -> 5^1;9^3;16^2
	4.138	0.5	16^1 -> 3^1;9^2;16^1

4.141	4.7	0 -> 5^1;8^1;9^1
4.142	3.9	0 -> 5^1;6^1
4.143	0.5	24^1 -> 5^2;24^1
4.143	0.6	0 -> 3^1;8^1
4.144	1.8	16^1 -> 5^1;6^1;16^1
4.146	0.8	16^2 -> 5^1;6^1;16^2
4.146	2.0	0 -> 7^2;9^1
4.147	0.8	11^1 -> 5^1;7^1;9^1;11^1

Table S6. Vibrational progression assignments for the absorption band I (3.7 - 4.6 eV) of 2-bromopyrimidine. From left to right, energies (in eV) of the experimental peak, and computed energies (in eV), intensities (in arbitrary units), and characters of the most important vibronic transitions between S_0 and S_1 electronic states. Several more transitions take place, however here we only show those with intensities larger than 1.0.

3.87	3.827	1.3	9^1 -> 0
	3.859	1.0	24^1 -> 24^1
	3.868	5.7	0 -> 0
	3.871	2.6	16^1 -> 16^1
	3.873	1.2	16^2 -> 16^2
	3.887	1.1	11^1 -> 9^1;11^1
3.91	3.899	1.6	24^1 -> 9^1;24^1
	3.909	8.5	0 -> 9^1
	3.912	3.9	16^1 -> 9^1;16^1
	3.914	1.8	16^2 -> 9^1;16^2
	3.940	1.2	24^1 -> 9^2;24^1
3.96	3.945	1.0	9^1 -> 7^1
	3.950	1.2	9^1 -> 9^3
	3.950	6.3	0 -> 9^2
	3.953	2.9	16^1 -> 9^2;16^1
	3.955	1.3	16^2 -> 9^2;16^2
	3.958	3.3	0 -> 8^1
	3.960	1.5	16^1 -> 8^1;16^1
	3.969	1.4	9^1 -> 5^1
4.00	3.987	4.1	0 -> 7^1
	3.989	1.9	16^1 -> 7^1;16^1
	3.991	1.1	9^1 -> 9^4
	3.991	3.1	0 -> 9^3
	3.994	1.4	16^1 -> 9^3;16^1
	3.999	4.7	0 -> 8^1;9^1
	4.000	3.9	0 -> 6^1
	4.001	1.1	24^1 -> 5^1;24^1
	4.001	2.2	16^1 -> 8^1;9^1;16^1
	4.002	1.8	16^1 -> 6^1;16^1
	4.003	1.0	16^2 -> 8^1;9^1;16^2
	4.010	5.8	0 -> 5^1
	4.013	2.7	16^1 -> 5^1;16^1
	4.015	1.2	16^2 -> 5^1;16^2

	4.018	1.1	24^1 -> 7^1;9^1;24^1
4.04	4.028	6.1	0 -> 7^1;9^1
	4.029	1.1	11^1 -> 5^1;9^1;11^1
	4.030	2.8	16^1 -> 7^1;9^1;16^1
	4.031	1.0	24^1 -> 6^1;9^1;24^1
	4.032	1.1	0 -> 9^4
	4.032	1.3	16^2 -> 7^1;9^1;16^2
	4.040	3.3	0 -> 8^1;9^2
	4.041	5.7	0 -> 6^1;9^1
	4.041	1.6	24^1 -> 5^1;9^1;24^1
	4.042	1.5	16^1 -> 8^1;9^2;16^1
	4.043	2.6	16^1 -> 6^1;9^1;16^1
	4.045	1.2	16^2 -> 6^1;9^1;16^2
	4.051	8.5	0 -> 5^1;9^1
	4.054	3.9	16^1 -> 5^1;9^1;16^1
	4.054	1.0	0 -> 3^1
	4.056	1.8	16^2 -> 5^1;9^1;16^2
4.08	4.069	4.5	0 -> 7^1;9^2
	4.071	2.1	16^1 -> 7^1;9^2;16^1
	4.076	2.3	0 -> 7^1;8^1
	4.079	1.1	16^1 -> 7^1;8^1;16^1
	4.081	1.5	0 -> 8^1;9^3
	4.081	4.1	0 -> 6^1;9^2
	4.082	1.1	24^1 -> 5^1;9^2;24^1
	4.084	1.9	16^1 -> 6^1;9^2;16^1
	4.087	1.0	9^1 -> 5^1;7^1
	4.088	1.2	0 -> 8^2;9^1
	4.089	2.2	0 -> 6^1;8^1
	4.091	1.0	16^1 -> 6^1;8^1;16^1
	4.092	1.1	9^1 -> 5^1;9^3
	4.092	6.2	0 -> 5^1;9^2
	4.095	2.8	16^1 -> 5^1;9^2;16^1
	4.095	1.5	0 -> 3^1;9^1
	4.097	1.3	16^2 -> 5^1;9^2;16^2
	4.100	1.0	9^1 -> 5^1;6^1
	4.100	3.4	0 -> 5^1;8^1
	4.102	1.5	16^1 -> 5^1;8^1;16^1
	4.105	1.4	0 -> 7^2
	4.110	2.1	0 -> 7^1;9^3

	4.112	1.0	16^1 -> 7^1;9^3;16^1
4.12	4.117	3.3	0 -> 7^1;8^1;9^1
	4.118	2.8	0 -> 6^1;7^1
	4.120	1.3	16^1 -> 6^1;7^1;16^1
	4.122	2.0	0 -> 6^1;9^3
	4.129	4.3	0 -> 5^1;7^1
	4.130	3.1	0 -> 6^1;8^1;9^1
	4.131	1.3	0 -> 6^2
	4.131	2.0	16^1 -> 5^1;7^1;16^1
	4.132	1.4	16^1 -> 6^1;8^1;9^1;16^1
	4.133	1.0	9^1 -> 5^1;9^4
	4.133	2.9	0 -> 5^1;9^3
	4.136	1.3	16^1 -> 5^1;9^3;16^1
	4.136	1.1	0 -> 3^1;9^2
	4.141	4.7	0 -> 5^1;8^1;9^1
	4.142	3.9	0 -> 5^1;6^1
	4.144	1.8	16^1 -> 5^1;6^1;16^1
	4.146	2.0	0 -> 7^2;9^1
	4.152	2.8	0 -> 5^2
	4.155	1.3	16^1 -> 5^2;16^1
	4.158	2.3	0 -> 7^1;8^1;9^2
4.17	4.159	4.1	0 -> 6^1;7^1;9^1
	4.160	1.1	24^1 -> 5^1;7^1;9^1;24^1
	4.170	6.2	0 -> 5^1;7^1;9^1
	4.171	2.1	0 -> 6^1;8^1;9^2
	4.172	1.8	0 -> 6^2;9^1
	4.173	1.0	24^1 -> 5^1;6^1;9^1;24^1
	4.174	1.0	0 -> 5^1;9^4
	4.182	3.3	0 -> 5^1;8^1;9^2
	4.183	5.6	0 -> 5^1;6^1;9^1
	4.187	1.5	0 -> 7^2;9^2
	4.193	4.0	0 -> 5^2;9^1
	4.196	1.8	16^1 -> 5^2;9^1;16^1
	4.199	1.1	0 -> 7^1;8^1;9^3
	4.200	2.9	0 -> 6^1;7^1;9^2
4.21	4.207	1.6	0 -> 6^1;7^1;8^1
	4.211	4.4	0 -> 5^1;7^1;9^2
	4.213	1.3	0 -> 6^2;9^2

	4.213	1.2	0 -> 3^1;7^1;9^1
	4.218	2.4	0 -> 5^1;7^1;8^1
	4.223	1.5	0 -> 5^1;8^1;9^3
	4.224	4.0	0 -> 5^1;6^1;9^2
	4.230	1.2	0 -> 5^1;8^2;9^1
	4.231	2.2	0 -> 5^1;6^1;8^1
	4.234	2.8	0 -> 5^2;9^2
	4.236	1.1	0 -> 7^2;8^1;9^1
	4.237	1.3	16^1 -> 5^2;9^2;16^1
	4.237	1.3	0 -> 3^1;5^1;9^1
	4.241	1.4	0 -> 6^1;7^1;9^3
	4.242	1.6	0 -> 5^2;8^1
4.25	4.247	1.4	0 -> 5^1;7^2
	4.248	2.2	0 -> 6^1;7^1;8^1;9^1
	4.252	2.1	0 -> 5^1;7^1;9^3
	4.259	3.3	0 -> 5^1;7^1;8^1;9^1
	4.260	2.9	0 -> 5^1;6^1;7^1
	4.264	1.8	0 -> 5^1;6^1;9^3
	4.271	2.1	0 -> 5^2;7^1
	4.272	3.1	0 -> 5^1;6^1;8^1;9^1
	4.273	1.3	0 -> 5^1;6^2
	4.275	1.3	0 -> 5^2;9^3
	4.277	1.3	0 -> 6^1;7^2;9^1
	4.283	2.2	0 -> 5^2;8^1;9^1
	4.284	1.9	0 -> 5^2;6^1
	4.288	2.1	0 -> 5^1;7^2;9^1
	4.289	1.5	0 -> 6^1;7^1;8^1;9^2
	4.290	1.3	0 -> 6^2;7^1;9^1
4.29	4.300	2.3	0 -> 5^1;7^1;8^1;9^2
	4.301	4.0	0 -> 5^1;6^1;7^1;9^1
	4.312	2.9	0 -> 5^2;7^1;9^1
	4.313	2.1	0 -> 5^1;6^1;8^1;9^2
	4.314	1.8	0 -> 5^1;6^2;9^1
	4.324	1.5	0 -> 5^2;8^1;9^2
	4.325	2.6	0 -> 5^2;6^1;9^1
	4.329	1.4	0 -> 5^1;7^2;9^2
4.34	4.335	1.2	0 -> 5^3;9^1
	4.341	1.0	0 -> 5^1;7^1;8^1;9^3

	4.342	2.8	0 -> 5^1;6^1;7^1;9^2
	4.349	1.6	0 -> 5^1;6^1;7^1;8^1
	4.353	2.0	0 -> 5^2;7^1;9^2
	4.355	1.2	0 -> 5^1;6^2;9^2
	4.356	1.0	0 -> 3^1;5^1;7^1;9^1
	4.360	1.2	0 -> 5^2;7^1;8^1
	4.366	1.8	0 -> 5^2;6^1;9^2
	4.373	1.1	0 -> 5^2;6^1;8^1
	4.378	1.1	0 -> 5^1;7^2;8^1;9^1
4.39	4.383	1.3	0 -> 5^1;6^1;7^1;9^3
	4.390	2.2	0 -> 5^1;6^1;7^1;8^1;9^1
	4.401	1.6	0 -> 5^2;7^1;8^1;9^1
	4.402	1.4	0 -> 5^2;6^1;7^1
	4.414	1.4	0 -> 5^2;6^1;8^1;9^1
	4.419	1.3	0 -> 5^1;6^1;7^2;9^1

Table S7. Vibrational progression assignments for the absorption band I (3.7 - 4.6 eV) of 5-bromopyrimidine. From left to right, energies (in eV) of the experimental peak, and computed energies (in eV), intensities (in arbitrary units), and characters of the most important vibronic transitions between S_0 and S_1 electronic states. Several more transitions take place, however here we only show those with intensities larger than 2.0.

3.79	3.768	2.3	11^1 -> 23^1
	3.785	3.1	16^2 -> 24^2
	3.786	7.9	16^1 -> 24^1
	3.787	2.7	9^1 -> 15^1
	3.787	2.4	24^1;16^1 -> 16^1;24^1
	3.787	20.1	0 -> 0
	3.788	6.0	24^1 -> 16^1
3.83	3.827	4.5	0 -> 15^1
	3.843	2.4	0 -> 17^2
	3.855	2.7	11^1 -> 14^1;23^1
3.87	3.873	3.7	16^2 -> 14^1;24^2
	3.874	9.3	16^1 -> 14^1;24^1
	3.874	3.1	9^1 -> 14^1;15^1
	3.874	2.8	24^1;16^1 -> 14^1;16^1;24^1
	3.875	3.2	16^1 -> 13^1;24^1
	3.875	23.7	0 -> 14^1
	3.875	7.0	24^1 -> 14^1;16^1
	3.876	2.1	24^2 -> 14^1;16^2
	3.876	8.0	0 -> 13^1
	3.876	2.4	24^1 -> 13^1;16^1
	3.889	2.3	11^1 -> 12^1;23^1
3.91	3.903	2.2	0 -> 13^1;17^1
	3.906	3.2	16^2 -> 12^1;24^2
	3.907	8.1	16^1 -> 12^1;24^1
	3.907	2.8	9^1 -> 12^1;15^1
	3.908	2.4	24^1;16^1 -> 12^1;16^1;24^1
	3.908	20.5	0 -> 12^1
	3.909	6.1	24^1 -> 12^1;16^1
	3.915	5.0	0 -> 14^1;15^1
	3.915	4.1	16^1 -> 11^1;24^1
	3.916	2.1	0 -> 13^1;15^1
	3.916	10.3	0 -> 11^1
	3.917	3.1	24^1 -> 11^1;16^1
	3.920	3.5	16^1 -> 10^1;24^1
	3.921	8.9	0 -> 10^1

	3.922	2.7	24^1 -> 10^1;16^1
	3.929	2.7	16^1 -> 9^1;24^1
	3.930	3.2	0 -> 14^1;17^2
	3.930	7.0	0 -> 9^1
	3.931	2.1	24^1 -> 9^1;16^1
	3.948	4.7	0 -> 12^1;15^1
3.96	3.956	2.3	0 -> 11^1;15^1
	3.961	4.9	16^1 -> 14^2;24^1
	3.962	4.5	16^1 -> 13^1;14^1;24^1
	3.962	12.5	0 -> 14^2
	3.963	3.7	24^1 -> 14^2;16^1
	3.963	2.4	0 -> 12^1;17^2
	3.963	11.5	0 -> 13^1;14^1
	3.964	3.4	24^1 -> 13^1;14^1;16^1
	3.976	2.7	11^1 -> 12^1;14^1;23^1
	3.981	4.2	0 -> 4^1
4.00	3.991	2.4	0 -> 13^1;14^1;17^1
	3.993	3.7	16^2 -> 12^1;14^1;24^2
	3.994	9.3	16^1 -> 12^1;14^1;24^1
	3.995	3.1	9^1 -> 12^1;14^1;15^1
	3.995	2.8	24^1;16^1 -> 12^1;14^1;16^1;24^1
	3.995	3.2	16^1 -> 12^1;13^1;24^1
	3.995	23.5	0 -> 12^1;14^1
	3.996	7.0	24^1 -> 12^1;14^1;16^1
	3.996	2.1	24^2 -> 12^1;14^1;16^2
	3.996	8.1	0 -> 12^1;13^1
	3.997	2.4	24^1 -> 12^1;13^1;16^1
	4.002	2.4	0 -> 14^2;15^1
	4.003	4.8	16^1 -> 11^1;14^1;24^1
	4.003	2.7	0 -> 13^1;14^1;15^1
	4.004	12.1	0 -> 11^1;14^1
	4.004	3.6	24^1 -> 11^1;14^1;16^1
	4.005	3.8	0 -> 11^1;13^1
	4.008	4.1	16^1 -> 10^1;14^1;24^1
	4.009	10.4	0 -> 10^1;14^1
	4.009	3.1	24^1 -> 10^1;14^1;16^1
	4.010	3.7	0 -> 10^1;13^1
	4.017	3.2	16^1 -> 9^1;14^1;24^1
	4.018	8.0	0 -> 9^1;14^1
	4.018	2.4	24^1 -> 9^1;14^1;16^1
	4.019	3.1	0 -> 9^1;13^1
	4.024	2.3	0 -> 12^1;13^1;17^1

	4.028	3.9	16^1 -> 12^2;24^1
	4.029	9.9	0 -> 12^2
	4.029	2.9	24^1 -> 12^2;16^1
4.04	4.035	4.9	0 -> 12^1;14^1;15^1
	4.036	4.2	16^1 -> 11^1;12^1;24^1
	4.036	2.1	0 -> 12^1;13^1;15^1
	4.037	10.6	0 -> 11^1;12^1
	4.037	3.2	24^1 -> 11^1;12^1;16^1
	4.041	3.6	16^1 -> 10^1;12^1;24^1
	4.042	9.1	0 -> 10^1;12^1
	4.042	2.7	24^1 -> 10^1;12^1;16^1
	4.043	2.5	0 -> 11^1;14^1;15^1
	4.048	2.1	0 -> 10^1;14^1;15^1
	4.050	2.8	16^1 -> 9^1;12^1;24^1
	4.050	2.9	16^1 -> 13^1;14^2;24^1
	4.050	3.8	0 -> 14^3
	4.050	4.9	0 -> 10^1;11^1
	4.051	3.2	0 -> 12^1;14^1;17^2
	4.051	7.1	0 -> 9^1;12^1
	4.051	7.3	0 -> 13^1;14^2
	4.051	2.1	24^1 -> 9^1;12^1;16^1
	4.051	2.2	24^1 -> 13^1;14^2;16^1
	4.059	3.8	0 -> 9^1;11^1
	4.064	2.9	0 -> 9^1;10^1
	4.068	5.0	0 -> 4^1;14^1
	4.068	2.2	0 -> 12^2;15^1
	4.077	2.4	0 -> 11^1;12^1;15^1
4.08	4.081	2.0	0 -> 10^1;12^1;15^1
	4.082	4.7	16^1 -> 12^1;14^2;24^1
	4.083	4.4	16^1 -> 12^1;13^1;14^1;24^1
	4.083	12.0	0 -> 12^1;14^2
	4.083	3.6	24^1 -> 12^1;14^2;16^1
	4.084	11.3	0 -> 12^1;13^1;14^1
	4.084	3.4	24^1 -> 12^1;13^1;14^1;16^1
	4.090	2.5	16^1 -> 11^1;14^2;24^1
	4.091	2.2	16^1 -> 11^1;13^1;14^1;24^1
	4.091	6.3	0 -> 11^1;14^2
	4.092	5.5	0 -> 11^1;13^1;14^1
	4.095	2.2	16^1 -> 10^1;14^2;24^1
	4.096	2.1	16^1 -> 10^1;13^1;14^1;24^1
	4.096	5.5	0 -> 10^1;14^2
	4.097	5.3	0 -> 10^1;13^1;14^1

	4.101	4.4	0 -> 4^1;12^1
	4.105	4.1	0 -> 9^1;14^2
	4.106	4.4	0 -> 9^1;13^1;14^1
	4.111	2.3	0 -> 12^1;13^1;14^1;17^1
	4.115	4.4	16^1 -> 12^2;14^1;24^1
	4.116	11.1	0 -> 12^2;14^1
	4.117	3.3	24^1 -> 12^2;14^1;16^1
	4.117	3.8	0 -> 12^2;13^1
4.12	4.123	2.3	0 -> 12^1;14^2;15^1
	4.123	4.8	16^1 -> 11^1;12^1;14^1;24^1
	4.124	2.7	0 -> 12^1;13^1;14^1;15^1
	4.124	12.1	0 -> 11^1;12^1;14^1
	4.125	3.6	24^1 -> 11^1;12^1;14^1;16^1
	4.125	3.9	0 -> 11^1;12^1;13^1
	4.128	4.1	16^1 -> 10^1;12^1;14^1;24^1
	4.129	10.3	0 -> 10^1;12^1;14^1
	4.130	3.1	24^1 -> 10^1;12^1;14^1;16^1
	4.130	3.7	0 -> 10^1;12^1;13^1
	4.133	2.2	0 -> 11^2;14^1
	4.137	2.3	16^1 -> 10^1;11^1;14^1;24^1
	4.137	3.1	16^1 -> 9^1;12^1;14^1;24^1
	4.138	5.7	0 -> 10^1;11^1;14^1
	4.138	8.0	0 -> 9^1;12^1;14^1
	4.138	2.8	0 -> 13^1;14^3
	4.139	2.4	24^1 -> 9^1;12^1;14^1;16^1
	4.139	3.2	0 -> 9^1;12^1;13^1
	4.142	2.0	0 -> 10^2;14^1
	4.146	4.4	0 -> 9^1;11^1;14^1
	4.149	3.0	0 -> 12^3
	4.151	3.4	0 -> 9^1;10^1;14^1
	4.155	2.7	0 -> 4^1;14^2
	4.156	2.3	0 -> 12^2;14^1;15^1
	4.156	2.2	0 -> 4^1;13^1;14^1
	4.157	2.0	16^1 -> 11^1;12^2;24^1
	4.158	5.2	0 -> 11^1;12^2
	4.162	4.3	0 -> 10^1;12^2
	4.164	2.5	0 -> 11^1;12^1;14^1;15^1
4.17	4.169	2.1	0 -> 10^1;12^1;14^1;15^1
	4.170	2.8	16^1 -> 12^1;13^1;14^2;24^1
	4.170	3.6	0 -> 12^1;14^3
	4.171	5.1	0 -> 10^1;11^1;12^1
	4.171	3.4	0 -> 9^1;12^2

	4.171	7.0	0 -> 12^1;13^1;14^2
	4.172	2.1	24^1 -> 12^1;13^1;14^2;16^1
	4.180	3.9	0 -> 9^1;11^1;12^1
	4.180	3.5	0 -> 11^1;13^1;14^2
	4.184	3.0	0 -> 9^1;10^1;12^1
	4.184	3.3	0 -> 10^1;13^1;14^2
	4.188	2.0	16^1 -> 4^1;12^1;14^1;24^1
	4.189	5.1	0 -> 4^1;12^1;14^1
	4.193	2.7	0 -> 9^1;13^1;14^2
	4.197	2.2	0 -> 4^1;11^1;14^1
	4.202	2.2	16^1 -> 12^2;14^2;24^1
	4.203	2.1	16^1 -> 12^2;13^1;14^1;24^1
	4.204	5.5	0 -> 12^2;14^2
	4.204	5.3	0 -> 12^2;13^1;14^1
4.21	4.211	2.4	16^1 -> 11^1;12^1;14^2;24^1
	4.212	2.1	16^1 -> 11^1;12^1;13^1;14^1;24^1
	4.212	6.2	0 -> 11^1;12^1;14^2
	4.213	5.4	0 -> 11^1;12^1;13^1;14^1
	4.216	2.1	16^1 -> 10^1;12^1;14^2;24^1
	4.217	2.0	16^1 -> 10^1;12^1;13^1;14^1;24^1
	4.217	5.3	0 -> 10^1;12^1;14^2
	4.218	5.1	0 -> 10^1;12^1;13^1;14^1
	4.222	2.2	0 -> 4^1;12^2
	4.225	3.0	0 -> 10^1;11^1;14^2
	4.226	4.0	0 -> 9^1;12^1;14^2
	4.226	2.7	0 -> 10^1;11^1;13^1;14^1
	4.227	4.3	0 -> 9^1;12^1;13^1;14^1
	4.234	2.2	0 -> 9^1;11^1;14^2
	4.235	2.2	0 -> 9^1;11^1;13^1;14^1
	4.237	3.2	0 -> 12^3;14^1
	4.244	2.3	16^1 -> 11^1;12^2;14^1;24^1
	4.245	5.8	0 -> 11^1;12^2;14^1
4.25	4.250	4.8	0 -> 10^1;12^2;14^1
	4.253	2.2	0 -> 11^2;12^1;14^1
	4.257	2.3	16^1 -> 10^1;11^1;12^1;14^1;24^1
	4.258	5.7	0 -> 10^1;11^1;12^1;14^1
	4.259	3.8	0 -> 9^1;12^2;14^1
	4.259	2.6	0 -> 12^1;13^1;14^3
	4.267	4.4	0 -> 9^1;11^1;12^1;14^1
	4.272	3.3	0 -> 9^1;10^1;12^1;14^1
	4.276	2.7	0 -> 4^1;12^1;14^2
	4.277	2.2	0 -> 4^1;12^1;13^1;14^1

	4.291	2.4	0 -> 10^1;11^1;12^2
	4.292	3.2	0 -> 12^2;13^1;14^2
4.29	4.300	3.4	0 -> 11^1;12^1;13^1;14^2
	4.305	3.2	0 -> 10^1;12^1;13^1;14^2
	4.309	2.5	0 -> 4^1;12^2;14^1
	4.314	2.6	0 -> 9^1;12^1;13^1;14^2
	4.317	2.3	0 -> 4^1;11^1;12^1;14^1
	4.332	2.9	0 -> 11^1;12^2;14^2
	4.333	2.6	0 -> 11^1;12^2;13^1;14^1
	4.337	2.4	0 -> 10^1;12^2;14^2
	4.338	2.4	0 -> 10^1;12^2;13^1;14^1
4.33	4.346	2.9	0 -> 10^1;11^1;12^1;14^2
	4.347	2.7	0 -> 10^1;11^1;12^1;13^1;14^1
	4.354	2.2	0 -> 9^1;11^1;12^1;14^2
	4.355	2.2	0 -> 9^1;11^1;12^1;13^1;14^1
	4.379	2.7	0 -> 10^1;11^1;12^2;14^1
4.38	4.388	2.1	0 -> 9^1;11^1;12^2;14^1
